# Global, Regional, and National Burden of Smoking-Related Diseases and Associations With Health Workforce Distribution, 1990–2021: Analysis From the Global Burden of Disease Study 2021

**DOI:** 10.3389/ijph.2025.1608217

**Published:** 2025-07-02

**Authors:** Yuzhou Cai, Guiming Chen, Peng Bai

**Affiliations:** ^1^ Department of Gastrointestinal Surgery, The First Affiliated Hospital of Kunming Medical University, Kunming, Yunnan, China; ^2^ Interventional Medicine Department, The First Affiliated Hospital of Kunming Medical University, Kunming, China

**Keywords:** smoking-related diseases, global health burden, health workforce, disease trends, prevention strategies

## Abstract

**Objectives:**

To analyze global trends in smoking-related disease burden from 1990–2021 and examine associations with health workforce distribution across countries.

**Methods:**

We analyzed smoking-related deaths and disability-adjusted life years using Global Burden of Disease 2021 data for 204 countries. Age-standardized rates were calculated for 27 geographic regions. Linear regression assessed temporal trends, while autoregressive integrated moving average models projected future burden to 2050. Correlation analyses examined relationships between 22 health workforce categories and disease burden.

**Results:**

Globally, age-standardized death rates from smoking-related diseases increased by 12.3% from 1990–2021, with males showing higher rates than females across all regions. Middle Socio-demographic Index regions exhibited the highest burden. Pharmaceutical technicians demonstrated strong positive correlations with disease burden (r = 0.35–0.37, p < 0.001), while traditional practitioners showed negative correlations (r = −0.24 to −0.28, p < 0.001). Projections indicate continued increases through 2050.

**Conclusion:**

Smoking-related disease burden demonstrates significant geographic and temporal variations, with distinct associations between health workforce composition and disease patterns, highlighting the need for targeted prevention strategies.

## Introduction

Tobacco use remains one of the most significant preventable causes of death and disability worldwide, representing a persistent global health challenge with profound societal implications. Recent genetic studies have identified 461 potential risk genes associated with tobacco dependence, underscoring the complex biological mechanisms underlying tobacco use disorder [1]. The epidemiological landscape continues to evolve, with traditional cigarette smoking prevalence declining in high-income countries while alternative tobacco products gain popularity. Notably, approximately 7.2 million adults in the United States transitioned to exclusive e-cigarette use between 2017 and 2023, maintaining overall tobacco use at concerning levels despite reductions in conventional smoking [2]. Among vulnerable populations, the burden is particularly severe; for instance, more than 40% of people with HIV smoke tobacco cigarettes, and smoking decreases life expectancy more than HIV itself among those receiving antiretroviral therapy [3].

Contemporary research has advanced our understanding of smoking-related disease burden through comprehensive epidemiological analyses and policy evaluations. Comparative studies suggest that e-cigarette use may present reduced disease risks compared to traditional smoking, with pooled odds ratios of 0.48 for myocardial infarction, 0.65 for stroke, and 0.46 for chronic obstructive pulmonary disease [4]. Policy interventions have demonstrated varying effectiveness, with systematic reviews indicating that taxation and multifaceted approaches can achieve meaningful reductions in smokeless tobacco use, ranging from 4.4% to 30.3% for taxation policies and 22.2%–70.9% for comprehensive strategies [5]. Additionally, significant disparities persist across populations, with healthcare provider screening reaching only 42.8% of youth, and notable differences observed across racial and ethnic groups [1].

Despite these advances, substantial research gaps remain in our understanding of global smoking-related disease burden. Previous studies have primarily focused on high-income countries or specific tobacco products, with limited comprehensive analyses examining temporal trends across diverse socioeconomic contexts. The relationship between health workforce distribution and smoking-related disease outcomes represents a particularly understudied area, despite its potential importance for developing targeted interventions. Furthermore, while individual risk factors have been extensively studied, the complex interplay between health system capacity, socioeconomic development, and disease burden patterns requires more systematic investigation to inform evidence-based policy decisions.

Gender and socioeconomic disparities add additional complexity to the global tobacco epidemic that requires continued investigation. Among older adults in India, tobacco use and food insecurity independently contribute to increased risk of underweight status, with smoking tobacco users being twice as likely to be underweight compared to non-users (OR = 2.07, 95% CI = 1.79–2.40) [6]. In low- and middle-income countries, while tobacco use among women remains relatively low at 3.2%, secondhand smoke exposure affects 23.0% of women, with pregnant women experiencing even higher exposure rates [7]. These patterns highlight the need for comprehensive analyses that capture the full spectrum of tobacco-related health impacts across different populations and developmental contexts.

Predictive modeling approaches offer valuable opportunities for anticipating future disease burden trends and informing long-term policy planning. Advanced methodological frameworks, including autoregressive integrated moving average (ARIMA) models and exponential smoothing techniques, enable researchers and policymakers to develop proactive strategies for mitigating smoking-related health impacts. However, limited studies have employed these approaches to generate comprehensive global projections that can guide resource allocation and intervention planning across diverse healthcare systems and socioeconomic contexts.

This study aims to address these critical knowledge gaps by providing a comprehensive assessment of global, regional, and national trends in smoking-attributable deaths and disability-adjusted life years from 1990 to 2021, examining novel associations between health workforce composition and disease outcomes, and generating robust projections for future burden through 2050. By integrating analyses of health workforce relationships with established epidemiological methods and employing advanced forecasting methodologies, this research contributes essential evidence for developing targeted, context-specific approaches to tobacco control and health system strengthening in the evolving global tobacco landscape.

## Methods

### Data Sources and Overview

Data on annual tobacco use cases and age-standardized prevalence were sourced from the GBD 2021 database, covering 23 age groups (birth to 95+ years), 204 countries and territories, 21 regions, and seven super-regions. Regions are defined by geographical and epidemiological similarities, while super-regions reflect shared patterns of cause-specific mortality. The Global Environmental and Development Report 2021 offers subnational insights for 21 select countries, with some results stratified by the socio-demographic index (SDI) [8]. Health workforce data were obtained from the Global Health Observatory of the World Health Organization, providing densities per 10,000 population for 22 health workforce categories across countries in 1990 and 2019. The standard practice in GBD methodology refers to setting mortality and remission rates to zero in the DisMod-MR framework when these parameters cannot be reliably estimated from available data sources, which is the established approach for risk factor attribution studies [9].

### Study Population and Data Processing

Data processing involved several components: (1) Age-standardized rates and uncertainty intervals were directly extracted from the GBD database; (2) Percentage changes in case numbers and age-standardized rates between 1990 and 2021 were calculated using the formula: [(value_2021_ - value_1990_)/value_1990_] × 100; (3) Estimated annual percentage change (EAPC) values were computed using linear regression models applied to natural logarithm-transformed age-standardized rates against calendar years, with EAPC = 100 × (e^β - 1), where β represents the regression coefficient.

### Statistical Analysis

We quantified deaths and DALYs attributable to smoking in 2021, alongside their age-standardized rates, stratified by age, sex, SDI, region, and country. Temporal trends from 1990 to 2021 were assessed using joinpoint regression analysis to identify significant changes in trend direction. Linear regression models were employed to calculate EAPC values, with statistical significance determined at p < 0.05.

Pearson correlation coefficients were calculated between health workforce densities and age-standardized disease rates for 204 countries in 1990 and 2019. Countries with extreme values (highest and lowest disease burden) were identified for detailed scatter plot analysis. Statistical significance was set at p < 0.05, with Bonferroni correction applied for multiple comparisons.

Future disease burden projections (2022–2050) employed autoregressive integrated moving average (ARIMA) models. Model selection followed a systematic approach: (1) Time series stationarity was assessed using the Augmented Dickey-Fuller test; (2) Model parameters (p, d, q) were determined using the auto. arima function in R, which employs Akaike Information Criterion (AIC) for optimal model selection; (3) Model assumptions were validated through residual analysis, including normality tests (Shapiro-Wilk) and autocorrelation assessment (Ljung-Box test); (4) Model performance was evaluated using in-sample mean absolute percentage error (MAPE) and out-of-sample validation with the last 5 years of data.

ARIMA model assumptions included: (1) stationarity of the differenced time series, (2) independence of residuals, (3) constant variance of residuals (homoscedasticity), and (4) normal distribution of residuals. Alternative exponential smoothing (ES) models were fitted for comparison, with final model selection based on forecast accuracy metrics.

All statistical analyses were conducted using R software (version 4.3.3), with specific packages including forecast (for ARIMA modeling), corrplot (for correlation visualization), and ggplot2 (for data visualization). Statistical significance was set at α = 0.05 for all analyses.

## Results

### The Disease Burden Attributable to Tobacco Use in 2021

In 2021, smoking-attributable deaths totaled 6,175,019 (with a 95% uncertainty interval, UI, of 5,047,661–7,226,385), accounting for a substantial 9.09% of all global deaths. The corresponding age-standardized death rate was 72.57 per 100,000 population (95% UI: 59.31–85.08), highlighting the pervasive impact of smoking on mortality worldwide. Furthermore, smoking-related DALYs amounted to 165,080,660 (95% UI: 135,430,162–193,938,448), constituting 5.73% of the global DALYs burden. The age-standardized DALY rate for [the specified condition/outcome] was 77.04 per 100,000 individuals (95% UI: 37.03–119.52), as detailed in Supplementary Tables S1, S2. These findings underscore the significant contribution of smoking to the global burden of disease and disability.

**FIGURE 1:**
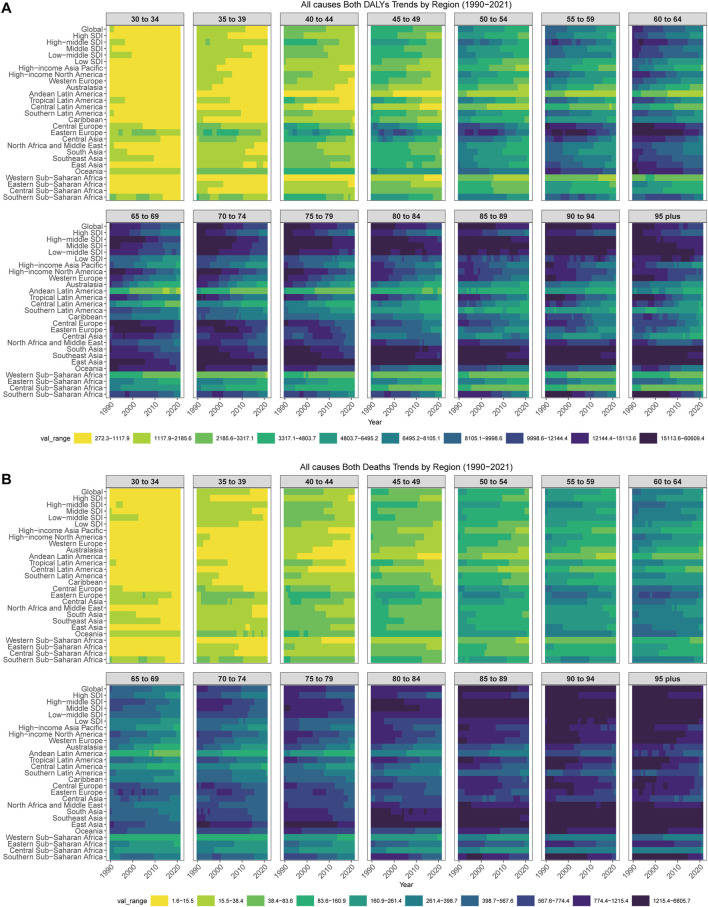
Age-specific rates of smoking-related diseases by global regions and Socio-demographic Index regions, 1990–2021. **(A)** Age-specific disability-adjusted life years rates of smoking-related diseases for both sexes across global regions and Socio-demographic Index regions from 1990 to 2021. Heat map shows rates per 100,000 population across 14 age groups (30–34 to 95+ years) for 27 geographic regions including global, 5 Socio-demographic Index regions, and 21 Global Burden of Disease regions. Color intensity represents rate magnitude with darker colors indicating higher rates. **(B)** Age-specific death rates of smoking-related diseases for both sexes across global regions and Socio-demographic Index regions from 1990 to 2021. Heat map shows rates per 100,000 population across 14 age groups (30–34 to 95+ years) for 27 geographic regions. Color intensity represents rate magnitude with darker colors indicating higher death rates. Data source: Global Burden of Disease Study 2021; coverage: global, regional, and national estimates; study period: 1990–2021.

Figure 1A depicts the distribution of deaths and DALYs across various age groups in 2021, providing a clear overview of the age-specific burden. Complementary to this, Figure 1B illustrates the trend in age-standardized death rates and age-standardized DALYs, revealing a consistent pattern of increase with advancing age, peaking in the 90–94 age bracket before declining. Notably, the trajectory of changes in the absolute number of deaths and DALY cases aligns with that of age-standardized DALYs, albeit with slightly earlier peaks observed in the 70–74 and 65–69 age groups, respectively (Figure 1; Supplementary Figure S1; Supplementary Tables S1, S2). This congruence underscores the robustness of the observed age-related patterns in health outcomes attributable to the studied factors.

At the level of SDI regions, our analysis revealed that the middle SDI regions experienced the greatest burden, with the highest number of smoking-related deaths at 2,073,568 cases and the highest number of DALYs at 54,276,330 cases. Additionally, the middle and high SDI regions demonstrated the highest age-standardized smoking rates, suggesting a notable correlation between socio-economic development and smoking prevalence (Figure 1; Supplementary Tables S1, S2). These findings underscore the importance of targeted interventions and policies to address smoking-related health disparities across different SDI regions.

Regarding gender-specific outcomes in 2021, our analysis demonstrated a pronounced disparity, with men experiencing 5.66 times more deaths and 5.59 times more DALYs compared to women. This gender gap was further underscored by the age-standardized rates, which were 6.88 times and 6.26 times higher for men in terms of deaths and DALYs, respectively (Figure 1; Supplementary Tables S1, S2). These findings emphasize the critical need for gender-tailored interventions and policies to mitigate the disproportionate burden of smoking-related harm borne by men.

**FIGURE 2:**
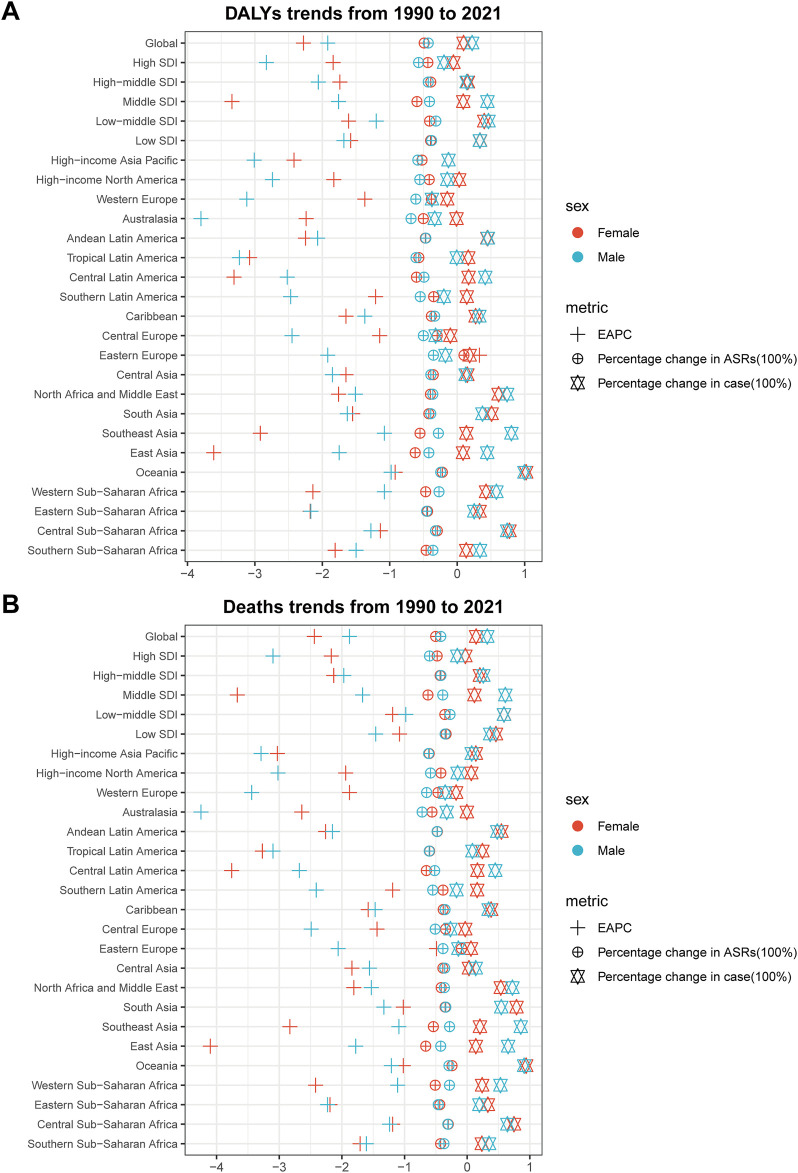
Trends in smoking-related diseases by sex across global regions and Socio-demographic Index regions, 1990–2021. **(A)** Trends in disability-adjusted life years of smoking-related diseases by sex across global regions and Socio-demographic Index regions, 1990–2021. Scatter plot displays percentage changes in cases (squares), percentage changes in age-standardized rates (diamonds), and estimated annual percentage changes (triangles) for males (blue) and females (red) across 27 geographic regions. **(B)** Trends in deaths from smoking-related diseases by sex across global regions and Socio-demographic Index regions, 1990–2021. Scatter plot displays percentage changes in cases (squares), percentage changes in age-standardized rates (diamonds), and estimated annual percentage changes (triangles) for males (blue) and females (red) across 27 geographic regions. Data source: Global Burden of Disease Study 2021; coverage: global, regional, and national estimates; study period: 1990–2021.

At the regional level within the GBD framework, studies have highlighted substantial disparities in the distribution of smoking-related health burdens. Specifically, Asia emerged as the leading region in terms of smoking-attributable deaths, accounting for a total of 4,303,522 deaths (95% uncertainty interval [UI]: 3,506,920–5,159,864). Concurrently, this region also bore the highest burden of smoking-related DALYs, totaling 111,107,808 DALYs (95% UI: 90,652,966–132,837,027). Notably, East Asia recorded the highest age-standardized mortality rate (115.03, 95% UI: 90.85–142.91), while Oceania stood out with a markedly elevated number of age-standardized DALYs (2908.95, 95% UI: 2180.25–3713.16), significantly surpassing other regions (Figure 2 and in Supplementary Tables S1, S2).

**FIGURE 3:**
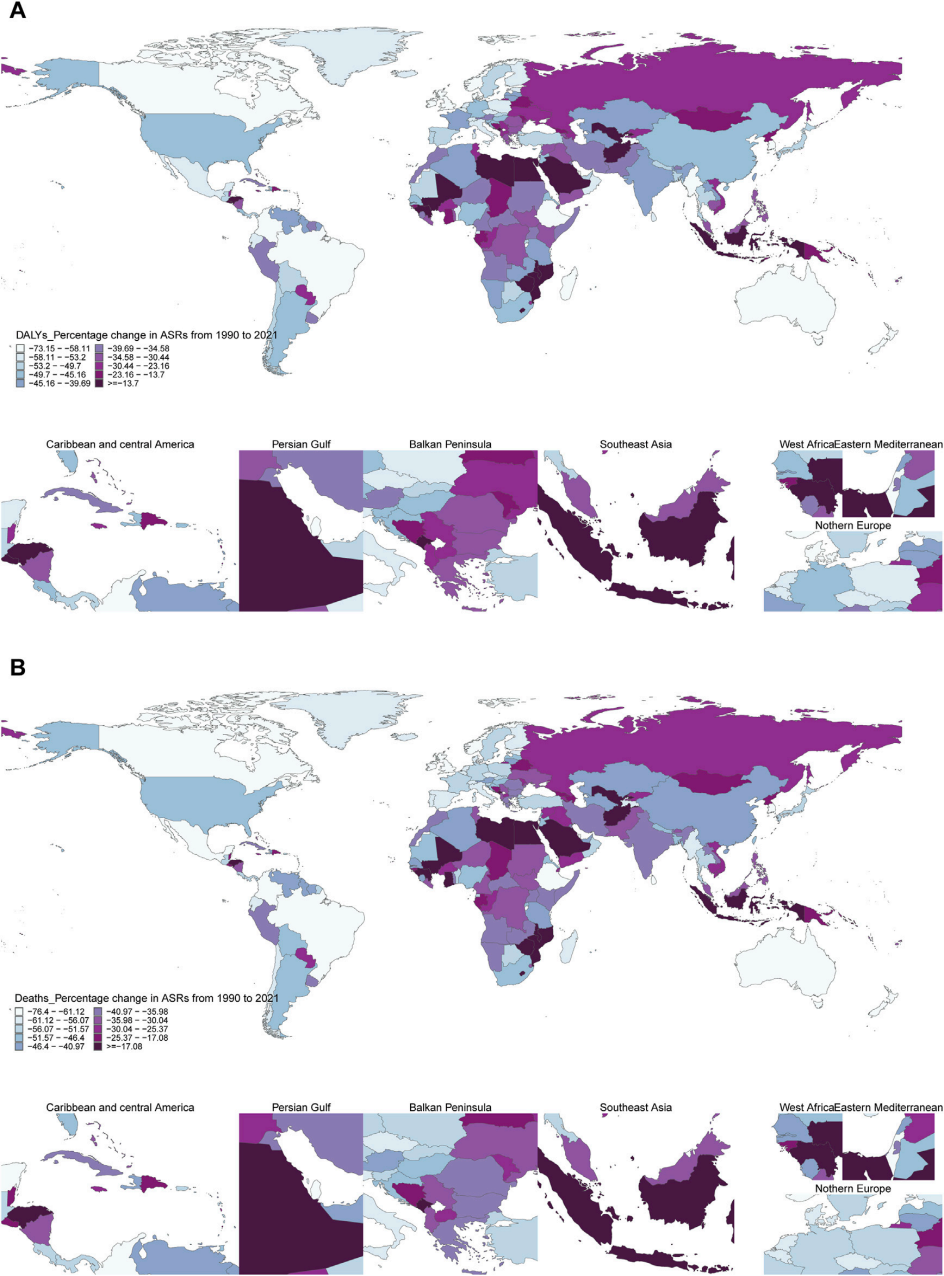
Global distribution of percentage changes in age-standardized rates of smoking-related diseases by country, 1990–2021. **(A)** Percentage change in age-standardized disability-adjusted life years rates of smoking-related diseases by country, 1990–2021. World map displays the percentage change in age-standardized disability-adjusted life years rates per 100,000 population for 204 countries and territories. Colors represent deciles of percentage change, with detailed regional insets for the Caribbean and Central America, Persian Gulf, Balkan Peninsula, Southeast Asia, West Africa, Eastern Mediterranean, and Northern Europe regions. Countries with insufficient data are shown in gray. **(B)** Percentage change in age-standardized death rates of smoking-related diseases by country, 1990–2021. World map displays the percentage change in age-standardized death rates per 100,000 population for 204 countries and territories. Colors represent deciles of percentage change, with detailed regional insets for seven key geographic regions. Countries with insufficient data are shown in gray. Data source: Global Burden of Disease Study 2021; coverage: global, regional, and national estimates; study period: 1990–2021.

Globally, the burden of disease attributable to smoking displays significant variation. In 2021, Kiribati stood out with the highest age-standardized mortality rate per 100,000 population (223.43, 95% uncertainty interval [UI]: 169.83–282.69), closely followed by Nauru and Lesotho. Similarly, Kiribati recorded the highest rate of age-standardized DALYs (6,888.85, 95% UI: 5,254.71–8,706.16), with Nauru and Micronesia (Federated States of) following closely behind. In contrast, Nigeria had the lowest age-standardized rates for both deaths and DALYs.However, it is noteworthy that China, despite not ranking among the top in age-standardized rates, experienced the highest observed number of smoking-related deaths (2,304,209; 95% UI: 1,814,317–2,896,305), surpassing India and the United States of America. This trend was mirrored in the number of DALYs, with China reporting the greatest burden (54,701,549, 95% UI: 43,108,563–68,748,854), followed by India and the United States. At the other end of the spectrum, the lowest numbers of smoking-related deaths and DALYs were observed in Tokelau and Niue, highlighting the substantial disparities in the global distribution of smoking-related health impacts (Figure 3; Supplementary Figure S2; Supplementary Tables S1, S2).

### Temporal Trend for Smoking-Related Disease Burden From 1990 to 2021

The global tally of smoking-related deaths has undergone a notable transformation, rising from 4,784,432 cases in 1990 to 6,175,019 cases in 2021, marking a substantial 29.08% increase over this period. Paradoxically, the corresponding age-standardized mortality rate has undergone an inverse trend, experiencing a decline of 42.46%. Similarly, the estimates of DALYs parallel this pattern, with the total number of DALYs cases rising by 20.03%, whereas the age-standardized DALYs rate has declined by 43.4% (Figure 3; Supplementary Tables S1, S2). The observed trends for males and females were consistent with those observed for the overall population. Moreover, the observed trends were consistent across the majority of age groups.

At the SDI region level, a uniform decline in age-standardized rates for smoking-related deaths and DALYs was observed across all SDI regions. Specifically, mortality rates exhibited varying trends within these regions: stabilization in low SDI regions, a decremental pattern in high SDI regions, a pronounced and sustained increase in medium SDI regions, a gradual upsurge in low-to-medium SDI regions, and an initial rise followed by a decline in medium-to-high SDI regions. With regards to DALYs, low SDI regions demonstrated a stable trend, whereas high SDI regions showed a decreasing trend. In contrast, medium SDI regions exhibited a notable and continuous increase in DALYs. Medium-low SDI regions also displayed a gradual increase, while medium-high SDI regions followed an initial increase that subsequently declined. These disparities underscore the multifaceted nature of the smoking epidemic and the need for tailored interventions across different SDI regions.

The burden of smoking-related disease manifests considerable variability across GBD regions. To discern regions with comparable shifts in disease burden, this study employed a stratified cluster analysis, with the findings presented in Figure 3. Notably, regions experiencing substantial increases in both age-standardized mortality and age-standardized DALYs include the High-Income Countries of the Commonwealth, Australasia, and Tropical Latin America. Conversely, regions demonstrating remarkable declines in these metrics encompass a diverse array, including high-income regions as categorized by the World Bank, Western Europe, the Americas, North America, particularly its high-income regions, advanced health system regions, East Africa, high-income regions within the Asia-Pacific, Central Latin America, as well as the Americas and the Caribbean (Figure 3; Supplementary Tables S1, S2). These findings highlight the diverse trends in smoking-related disease burden across GBD regions.

A comprehensive comparative analysis spanning 1990 to 2021 mortality data reveals that Saudi Arabia witnessed the most pronounced increase in smoking-related deaths, with a staggering rate of 244.33%. This surge was closely followed by the United Arab Emirates and Djibouti. In parallel, Ukraine emerged as the country with the most significant rise in DALYs, experiencing a remarkable 601% increase. The United Arab Emirates and Qatar followed suit, ranking second and third respectively, with increases of 24% and 23% in DALYs.Conversely, Estonia experienced the most substantial decrease, with a notable 49.78% reduction in deaths and a corresponding 52.42% decline in DALYs. This downward trend was also observed in the United Kingdom, albeit to a lesser extent. When examining age-standardized smoking rates and their impact on deaths and DALYs over the same period, Lesotho stands out for its notable increase, with an Estimated Annual Percentage Change (EAPC) of 2.21 (95% CI 1.80–2.26) for deaths and 2.51 (95% CI 2.06–2.09) for DALYs. On the other hand, Ireland demonstrated the most substantial decline in age-standardized mortality rates, with a DAPC of −4.59 (95% CI = −4.76 to −4.41) for DALYs. Meanwhile, the Maldives recorded the largest reduction in age-standardized DALYs, with an EAPC of −4.22 (95% CI = −4.47 to −3.97). These findings are visually represented in Figure 3, and are further detailed in Supplementary Tables S1, S2.

### The Predicted Results From 2022 to 2050

**FIGURE 4:**
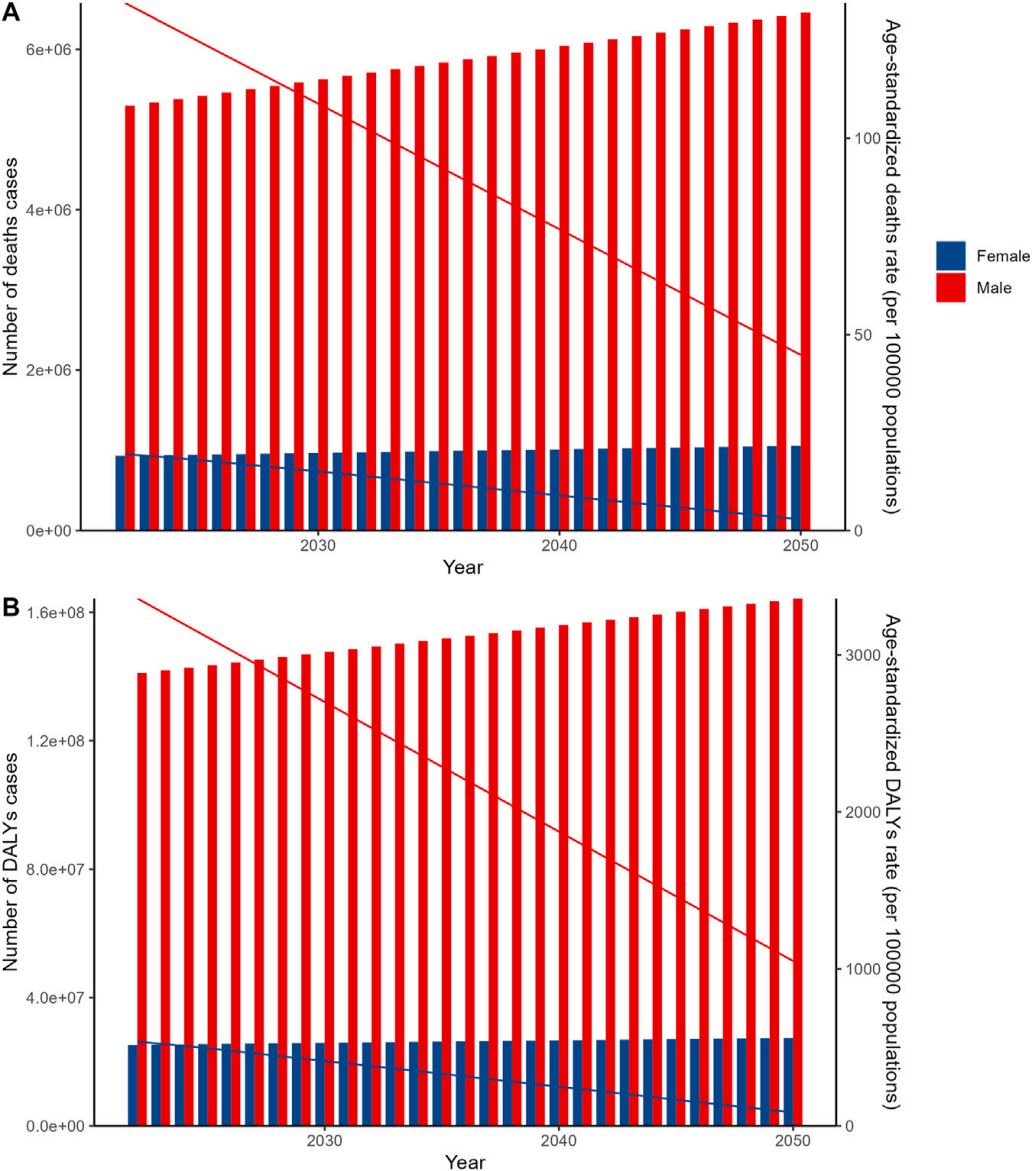
Autoregressive integrated moving average (ARIMA) model projections of global smoking-related diseases by sex, 2022–2050. **(A)** Autoregressive integrated moving average (ARIMA) model projections of global deaths from smoking-related diseases by sex and age-standardized death rates by sex, 2022–2050. **(B)** Autoregressive integrated moving average (ARIMA) model projections of global disability-adjusted life years from smoking-related diseases by sex and age-standardized disability-adjusted life years rates by sex, 2022–2050. Data source: Global Burden of Disease Study 2021; coverage: global, regional, and national estimates; study period: 1990–2021.

The projections derived from the ARIMA model predict an increase in the number of deaths and DALYs cases for both sexes from 2022 to 2050. Notably, despite this anticipated rise in absolute numbers, the corresponding age-standardized rates are projected to exhibit a declining trend. This pattern of results is mirrored by the outcomes from the ES model, providing convergent evidence and reinforcing the robustness of the findings (Figure 4; Supplementary Figure S3).

### Association Between Health Workforce Density and Smoking-Related Disease Burden

**FIGURE 5:**
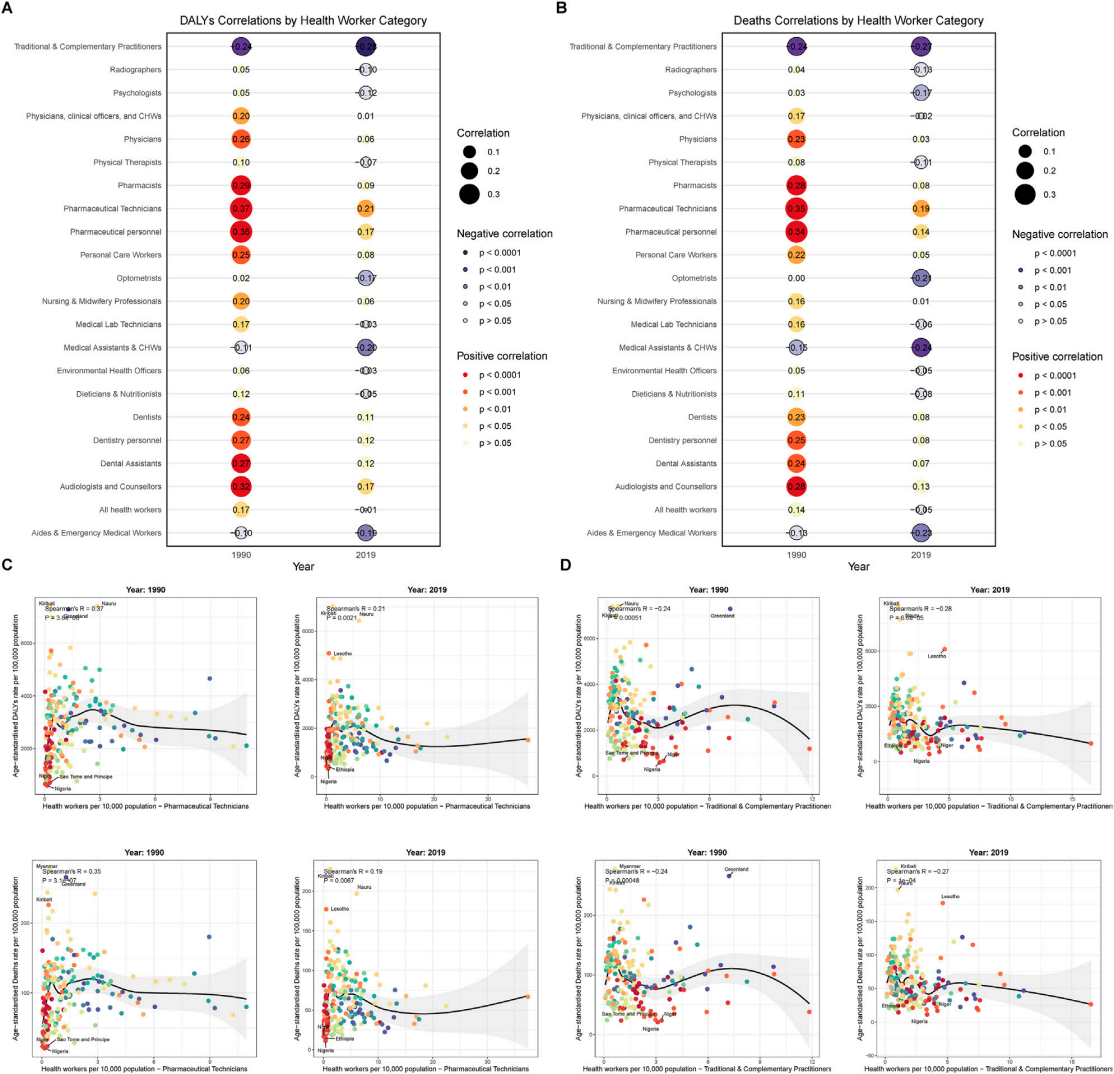
Correlation between health workforce density and smoking-related disease burden across countries. **(A)** Overall correlation patterns between health workforce categories and smoking-related disease burden, 1990 and 2019. Heat map displays correlation coefficients between 22 health workforce categories and smoking-related deaths and disability-adjusted life years rates across countries for both time periods. Red colors indicate positive correlations, blue colors indicate negative correlations, with color intensity representing correlation strength. **(B)** Overall correlation patterns between health workforce categories and smoking-related deaths across countries, 1990 and 2019. Heat map showing correlation coefficients specifically for death rates, with separate panels for 1990 and 2019 data. **(C)** Correlation between pharmaceutical technicians density and smoking-related disease burden across countries, 1990 and 2019. Scatter plots show the relationship between pharmaceutical technicians per 10,000 population and disability-adjusted life years rates (upper panels) and death rates (lower panels) of smoking-related diseases for 1990 (left) and 2019 (right). Linear regression lines with 95% confidence intervals are displayed. Correlation coefficients and p-values are indicated. **(D)** Correlation between traditional and complementary practitioners density and smoking-related disease burden across countries, 1990 and 2019. Scatter plots show the relationship between traditional and complementary practitioners per 10,000 population and disability-adjusted life years rates (upper panels) and death rates (lower panels) of smoking-related diseases for 1990 (left) and 2019 (right). Linear regression lines with 95% confidence intervals are displayed. Correlation coefficients and p-values are indicated. Data source: Global Burden of Disease Study 2021; coverage: global, regional, and national estimates; study period: 1990–2021.

To examine the relationship between health workforce distribution and smoking-related disease burden across countries, we conducted correlation analyses between 22 health workforce categories and age-standardized rates of smoking-related deaths and disability-adjusted life years (DALYs) for 204 countries and territories in 1990 and 2019 (Figures 5A,B). The analysis revealed distinct temporal patterns, with pharmaceutical technicians showing the strongest positive correlations in 1990 for both deaths (r = 0.35, p = 3.10 × 10^−7^) and DALYs (r = 0.37, p = 3.80 × 10^−8^), though these associations weakened by 2019 (deaths: r = 0.19, p = 0.0067; DALYs: r = 0.21, p = 0.0021). Conversely, traditional and complementary practitioners demonstrated consistent negative correlations that strengthened over time, with associations in 1990 (deaths: r = −0.24, p = 0.00048; DALYs: r = −0.24, p = 0.00051) becoming more pronounced by 2019 (deaths: r = −0.27, p = 1.00 × 10^−4^; DALYs: r = −0.28, p = 6.60 × 10^−5^). Other notable patterns included pharmaceutical personnel showing strong positive correlations in 1990 (deaths: r = 0.34, p = 6.30 × 10^−7^; DALYs: r = 0.36, p = 2.00 × 10^−7^) that substantially decreased by 2019, while medical assistants and community health workers exhibited strengthening negative correlations over the study period, suggesting complex relationships between health workforce composition and smoking-related disease burden across different healthcare system contexts. Detailed scatter plot analyses further illustrated these association patterns for the two extreme categories (Figures 5C,D), where countries with higher pharmaceutical technician densities (such as Nauru with 6.08 per 10,000 population in 2019, Kiribati with 1.22 per 10,000 in 2019) demonstrated higher disease rates (196.9 and 227.9 per 100,000 respectively), while countries with higher traditional and complementary practitioner densities (such as Greenland with 7.21 per 10,000 in 1990, Lesotho with 4.63 per 10,000 in 2019) tended to exhibit relatively lower disease burden, further supporting the complex associations between different health workforce configurations and smoking-related disease epidemiological characteristics.

## Discussion

To the best of our knowledge, this study represents the pioneering effort to comprehensively evaluate and quantify the global burden of disease attributable to tobacco use, while also projecting future trends in this burden. Our analysis underscores the substantial toll that tobacco consumption imposes on global health in 2021, with marked disparities observed across sexes, age groups, SDI regions, GBD regions, and individual countries. While there has been a discernible trend towards improvement in the global age-standardized rates (ASR) of tobacco-related disease from 1990 to 2021, it is concerning that the absolute number of deaths and DALYs remains alarmingly high and continues to escalate. Furthermore, our projections portend a persistent upward trajectory for both deaths and DALYs attributable to tobacco use over the subsequent 29 years. This underscores the urgent need for intensified global efforts to curb tobacco consumption and mitigate its devastating impact on public health.

The Global Adult Tobacco Survey (GATS) highlights tobacco use as a significant driver of global morbidity and mortality [10]. In Thailand, tobacco consumption and related mortality remain particularly high [11]. In 2018, 2 million new lung cancer cases and 1.7 million deaths were reported, largely linked to tobacco use [12]. Tobacco also strongly correlates with pancreatic cancer, with active smokers showing markedly higher mortality rates than non-smokers [13]. In Portuguese-speaking nations, tobacco was a key contributor to cardiovascular disease and premature deaths in 2019 [14]. Furthermore, tobacco use increases the risk of severe infectious respiratory diseases, as current smokers are more vulnerable to pneumonia and influenza [15]. Acrolein, a toxic component of tobacco smoke, plays a critical role in lung disease pathogenesis and progression to respiratory cancers [16]. These findings underscore the urgent need to address tobacco use as a major public health challenge. These findings underscore the urgent need to address tobacco use as a major public health challenge.

Long-term exposure to tobacco smoke has been incontrovertibly associated with an elevated risk of a myriad of tobacco-related illnesses, encompassing lung cancer, chronic obstructive pulmonary disease (COPD), and cardiovascular disease [17]. Notably, even minimal tobacco consumption has been demonstrated to exert a notable influence on cardiovascular health, highlighting the sensitivity of this system to tobacco’s harmful effects. Cessation of tobacco use, on the other hand, has been shown to swiftly diminish the likelihood of developing cardiovascular disease and to markedly decrease mortality rates related to coronary heart disease [18]. This underscores the importance of promoting smoking cessation strategies as an effective means of mitigating the cardiovascular burden imposed by tobacco. Furthermore, women who are exposed to secondhand smoke over prolonged periods, both domestically and occupationally, face a significantly heightened risk of developing lung cancer. The magnitude of this risk is intimately tied to the duration and intensity of exposure to secondhand smoke [19]. These observations serve as a stark reminder of the profound and enduring impact of tobacco use and exposure on human health, thereby emphasizing the urgent need for a current, comprehensive, and global assessment of the tobacco epidemic.

From 1990 to 2021, the decline in age-standardized burden reflects global expansion of tobacco control measures, with systematic reviews demonstrating that taxation and multifaceted policies achieve meaningful reductions in smokeless tobacco use ranging from 4.4% to 30.3% for taxation and 22.2%–70.9% for comprehensive approaches [5]. However, cessation service users showed increased healthcare utilization patterns, which may reflect underlying health conditions motivating cessation attempts rather than treatment success [20]. The emergence of e-cigarettes presents both opportunities and challenges, with comparative analyses showing reduced disease risks (OR: 0.48 for myocardial infarction, 0.65 for stroke, 0.46 for COPD) compared to traditional smoking [4]. The World Health Organization Framework Convention on Tobacco Control (FCTC) has been instrumental in boosting legal support for comprehensive tobacco control [21]. In the Netherlands, stringent policies significantly reduced the visibility and accessibility of tobacco products, highlighting their effectiveness [22]. E-cigarettes have also shown greater efficacy than nicotine replacement therapy in promoting smoking cessation, underscoring their potential in harm reduction strategies [23]. Additionally, research confirms that comprehensive policies, such as advertising bans and higher tobacco taxes, have delivered significant results in low- and middle-income countries [24]. These findings underscore the importance of evidence-based, multifaceted approaches in combating the global tobacco epidemic and improving public health.

Significant disparities exist in tobacco-related morbidity between men and women, with our findings showing men experiencing 5.66 times more deaths and 5.59 times more DALYs compared to women. This aligns with evidence from Latinx youth studies showing that children of non-immigrants had higher tobacco use prevalence, with these disparities persisting into young adulthood [25]. Additionally, among older populations, tobacco use compounds other health risks, as demonstrated in India where smoking tobacco users were twice as likely to be underweight compared to non-users (OR = 2.07, 95% CI = 1.79–2.40) [6]. Partly due to physiological differences, as estrogen offers some protection against tobacco smoke, particularly in premenopausal women [26]. Men are also more prone to conditions like hypertension, hyperlipidemia, and diabetes, which heighten the risk of severe tobacco-related illnesses [27]. Additionally, men tend to smoke more cigarettes daily and for longer durations, significantly increasing their disease burden [28]. These disparities highlight the need for public health policies tailored to men’s unique risks, aiming to reduce tobacco-related harms and improve health outcomes worldwide.

Moreover, age emerges as a pivotal variable that necessitates consideration, as individuals of advanced years are notably more susceptible to the deleterious consequences of tobacco consumption [29]. A protracted history of tobacco use translates into a heightened cumulative exposure to the noxious components present in tobacco products, compared to younger counterparts. Consequently, this cumulative exposure escalates the likelihood of adverse health outcomes [30]. As individuals advance in age, the natural decline in physiological functions undermines the body’s ability to mend the damage inflicted by tobacco smoke, thereby intensifying the risk of developing chronic obstructive pulmonary disease (COPD), lung cancer, and cardiovascular diseases [31]. Furthermore, empirical evidence underscores that tobacco use undermines the integrity of the immune system, rendering the elderly population more vulnerable to respiratory infections and other tobacco-related maladies [32]. These findings underscore the urgency for reinforcing health protection strategies specifically tailored to the elderly, aiming to mitigate the harmful effects of tobacco consumption in this vulnerable demographic.

The prevalence of tobacco-related diseases exhibits marked regional and national disparities, which can be traced to diverse environmental contexts, healthcare system capacities, and varying levels of economic development. High-income countries, exemplified by the United States, boast advanced healthcare systems; however, high smoking prevalence among specific demographics continues to impose substantial disease burdens, underscoring the complexity of addressing this issue [33]. Additionally, cultural norms and social acceptance of smoking play pivotal roles in shaping smoking rates, highlighting the need to consider sociocultural factors in intervention strategies [34]. In regions with a non-high SDI, the detrimental health impacts of smoking may be compounded by environmental and occupational hazards, including air pollution and exposure to industrial chemicals, often arising from rapid economic growth [35]. This underscores the need for a multifaceted approach that addresses both smoking behavior and the broader environmental determinants of health. Moreover, the implementation of tobacco control policies in medium SDI regions may lack the rigor and effectiveness observed in high SDI regions, potentially contributing to rising smoking rates and associated health complications [36]. This observation highlights the importance of tailored policy interventions that consider the unique challenges and resources available in different contexts, aiming to achieve more equitable outcomes in tobacco control efforts globally.

The findings of our projections reveal that from 2021 to 2050, both sexes will experience varying degrees of increase in mortality and DALYs, whereas the corresponding ASR are anticipated to remain relatively stagnant. This trend could be a consequence of demographic transitions, escalating high-risk behaviors, and socioeconomic advancements in the forthcoming decades. Consequently, we recommend integrating tertiary prevention measures into early health interventions to mitigate the disease burden. It is crucial to acknowledge several inherent limitations of this study. First, our reliance on the GBD database limits access to comprehensive data at finer geographical scales, such as counties, provinces, and states. Second, healthcare provider screening disparities may affect disease detection and reporting accuracy, as only 42.8% of youth receive tobacco use screening with notable racial and ethnic differences [1]. Third, the complex relationships between tobacco use and comorbid conditions, such as the 11–14 times higher odds of cannabis use among tobacco users in low- and middle-income countries [37], may influence disease burden estimates but are not fully captured in our analysis.

In conclusion, the projections presented herein are premised on the assumption of constancy in other influencing factors over the next three decades. However, it is imperative to recognize that some variables are inherently dynamic and may undergo changes over time. Given these limitations, future research endeavors should prioritize refining data collection methodologies and incorporating novel variables that could potentially influence disease burden projections. By securing more comprehensive and precise data, we can gain a deeper insight into regional and national disparities, ultimately facilitating the design and implementation of more tailored and effective interventions.

### Conclusion

The burden of disease from tobacco use remains a significant public health challenge, with stark disparities in mortality, morbidity, and age-standardized rates across nations. From 1990 to 2021, projections indicate an overall rise in tobacco-related disease burden globally, except in high-SDI regions. This trend reflects the complex interplay of country-specific factors and shifting epidemiological landscapes. Despite gradual improvements in equity, low- and middle-SDI countries continue to bear a disproportionate burden, driven by rising tobacco consumption and widening global disparities. Addressing these challenges requires innovative public health strategies alongside strong economic, pollution control, and environmental policies. Targeted interventions and adaptive measures are essential to strengthen public health systems, mitigate tobacco’s impact, and promote equitable healthcare access worldwide.

## Data Availability

All GBD data for this study are publicly available and can be found here: Global Burden of Disease (GBD) (healthdata.org).

## Supplementary Material

SUPPLEMENTARY FIGURE S1 Age-grouped global trends in smoking-related mortality, disability-adjusted life years (DALYs), and age-standardized rates. Abbreviations: DALYs, disability-adjusted life years.

SUPPLEMENTARY FIGURE S2 Results of cluster analysis based on the EAPC values of the tobacco-related age-standardized rates for deaths and DALYs from 1990 to 2021. Abbreviations: EAPC, estimated annual percentage change; DALYs, disability-adjusted life years.

SUPPLEMENTARY FIGURE S3 ES model projections of global tobacco-related deaths and disability-adjusted life years (DALYs) by sex and age-standardized mortality rates and age-standardized disability-adjusted life years (DALYs) by sex, 2022–2050. Abbreviations: DALYs, disability-adjusted life years; ES, exponential smoothing model.
