# Supplementary material for: Global, Regional, and National Burden of Smoking-Related Diseases and Associations With Health Workforce Distribution, 1990–2021: Analysis From the Global Burden of Disease Study 2021
Source: Int J Public Health. 2025 Jul 2;70:1608217. doi: 10.3389/ijph.2025.1608217 (PMC12263451; doi:10.3389/ijph.2025.1608217)
Supplement: Supplementary file 1 [file Table1.docx]

Table S1 Global number of cases and age-standardized death rates due to tobacco use in 1990 and 2021, and trends from 1990 to 2021.

| Characteristic | 1990 | | 2021 | | 1990-2021 |
| --- | --- | --- | --- | --- | --- |
|  | Number of deaths cases (95% UI) | The age-standardized deaths rate/100000（95%UI） | Number of deaths cases（95% UI） | The age-standardized deaths  rate/100000 (95% UI) | EAPC (95%CI) |
| **Global** | 4784432 (4087944-5484663) | 126.15 (107.1-145.41) | 6175019 (5047661-7226385) | 72.57 (59.31-85.08) | -1.91 (-1.98--1.84) |
| **Sex** |  |  |  |  |  |
| Female | 811872 (652794-988662) | 40.09 (32.15-49.05) | 927602 (739482-1160369) | 19.91 (15.88-24.95) | -2.44 (-2.55--2.33) |
| Male | 3972560 (3404000-4511921) | 237.06 (202.53-271.37) | 5247417 (4323258-6146650) | 137.08 (112.6-161.03) | -1.88 (-1.94--1.82) |
| **Age** |  |  |  |  |  |
| <30 years | 0 (0-0) | 0 (0-0) | 0 (0-0) | 0 (0-0) | 0 (0-0) |
| 30-34 years | 55953 (46963-65650) | 14.52 (12.18-17.03) | 44276 (35998-52605) | 7.32 (5.96-8.7) | -2.34 (-2.49--2.2) |
| 35-39 years | 93344 (79096-108074) | 26.5 (22.45-30.68) | 72392 (59792-84709) | 12.91 (10.66-15.1) | -2.52 (-2.64--2.4) |
| 40-44 years | 148521 (127703-170065) | 51.84 (44.58-59.36) | 16.91 | 25.65 |  |
| 45-49 years | 210211 (182325-240347) | 90.53 (78.52-103.51) | 208641 (174393-243369) | 44.06 (36.83-51.4) | -2.42 (-2.6--2.25) |
| 50-54 years | 356200 (309885-406576) | 167.57 (145.78-191.27) | 362709 (303945-426542) | 81.52 (68.31-95.87) | -2.42 (-2.54--2.29) |
| 55-59 years | 488838 (422846-555554) | 263.95 (228.32-299.98) | 542806 (455344-637159) | 137.17 (115.06-161.01) | -2.13 (-2.18--2.07) |
| 60-64 years | 655767 (567117-746692) | 408.3 (353.1-464.91) | 683338 (574926-792213) | 213.51 (179.64-247.53) | -2.14 (-2.2--2.09) |
| 65-69 years | 710015 (609413-808381) | 574.4 (493.01-653.98) | 865686 (719350-1018525) | 313.83 (260.78-369.24) | -2.15 (-2.23--2.07) |
| 70-74 years | 689174 (585099-790541) | 814.04 (691.11-933.77) | 939755 (774311-1117924) | 456.55 (376.17-543.11) | -2.04 (-2.12--1.96) |
| 75-79 years | 617727 (520010-722461) | 1003.53 (844.78-1173.67) | 807598 (660732-962978) | 612.35 (500.99-730.17) | -1.73 (-1.8--1.65) |
| 80-84 years | 418675 (342056-500222) | 1183.5 (966.92-1414.02) | 683288 (548789-824009) | 780.16 (626.59-940.83) | -1.44 (-1.54--1.35) |
| 85-89 years | 238333 (186985-290720) | 1577.21 (1237.4-1923.89) | 529503 (421280-648939) | 1158.1 (921.4-1419.32) | -1.19 (-1.35--1.02) |
| 90-94 years | 82488 (63889-101237) | 1924.95 (1490.92-2362.48) | 241965 (189352-296328) | 1352.56 (1058.46-1656.45) | -1.21 (-1.32--1.11) |
| 95+ years | 19187 (14549-24057) | 1884.64 (1429.03-2363.01) | 64772 (48554-81184) | 1188.42 (890.85-1489.53) | -1.55 (-1.65--1.45) |
| **SDI region** |  |  |  |  |  |
| High-middle SDI | 1348714 (1165834-1544152) | 141.15 (121.56-162.12) | 1685248 (1378931-2006555) | 85.39 (69.79-101.65) | -1.87 (-2.06--1.68) |
| High SDI | 1212588 (1039582-1393778) | 109.59 (93.98-125.98) | 1061570 (877857-1248889) | 49.31 (41.01-57.55) | -2.68 (-2.72--2.63) |
| Low-middle SDI | 699712 (564997-840501) | 122.3 (98.07-147.3) | 1109898 (902234-1325609) | 83.04 (67.21-99.91) | -1.18 (-1.24--1.12) |
| Low SDI | 173226 (137177-215268) | 82.39 (64.75-102.35) | 239643 (186869-295023) | 52.39 (40.53-65.17) | -1.46 (-1.56--1.36) |
| Middle SDI | 1344796 (1128627-1580893) | 145.82 (121.74-171.38) | 2073568 (1674749-2494309) | 82.17 (65.98-98.6) | -1.98 (-2.04--1.92) |
| **GBD region** |  |  |  |  |  |
| Advanced Health System | 1816736 (1572020-2076845) | 111.79 (96.68-127.88) | 1546493 (1288095-1813506) | 54.25 (45.48-63.21) | -2.54 (-2.67--2.41) |
| Africa | 179334 (146408-217550) | 65.99 (53.52-80.09) | 273480 (214047-332873) | 43.55 (34.3-53.09) | -1.41 (-1.49--1.33) |
| African Region | 123120 (98248-151613) | 57.39 (45.54-70.84) | 168989 (132531-205896) | 33.99 (26.78-41.63) | -1.85 (-1.95--1.74) |
| America | 623986 (531032-721635) | 103.06 (87.63-119.23) | 631069 (518622-754412) | 46.42 (38.2-55.43) | -2.74 (-2.81--2.67) |
| Andean Latin America | 7583 (6008-9231) | 38.01 (30.12-46.28) | 11307 (8603-14723) | 19.43 (14.78-25.27) | -2.2 (-2.4--2) |
| Asia | 2752102 (2309084-3231546) | 152.43 (127.15-179.33) | 4303522 (3506920-5159864) | 89.08 (72.22-106.83) | -1.82 (-1.87--1.76) |
| Australasia | 19828 (16602-23156) | 83.56 (69.87-97.64) | 15316 (12326-18758) | 28 (22.73-34.02) | -3.6 (-3.71--3.49) |
| Basic Health System | 2070388 (1735758-2440231) | 157.68 (131.6-185.6) | 3278174 (2643009-3950753) | 92.63 (74.53-111.75) | -1.82 (-1.9--1.74) |
| Caribbean | 19869 (16640-23394) | 78.61 (65.55-92.91) | 26860 (21344-32364) | 49.57 (39.39-59.73) | -1.53 (-1.6--1.46) |
| Central Africa | 14470 (11129-18350) | 50.22 (38.5-63.66) | 21493 (15957-27509) | 30.18 (22.65-38.67) | -1.66 (-1.75--1.57) |
| Central Asia | 48629 (41810-55434) | 102.29 (87.67-117.39) | 55022 (45382-64123) | 68.37 (56.31-80.03) | -1.46 (-1.75--1.17) |
| Central Europe | 211978 (183788-239447) | 142.95 (123.48-161.97) | 164463 (137342-190846) | 75.45 (63.26-87.31) | -2.22 (-2.3--2.15) |
| Central Latin America | 46866 (38607-55258) | 60.78 (49.69-72.09) | 64577 (51384-78325) | 26.23 (20.8-31.9) | -3.03 (-3.16--2.9) |
| Central Sub-Saharan Africa | 13000 (9843-16611) | 55.4 (41.84-70.37) | 21457 (15657-27730) | 36.21 (26.88-47.1) | -1.37 (-1.55--1.2) |
| Commonwealth High Income | 199911 (170533-229203) | 130.55 (111.37-149.23) | 125484 (102823-149988) | 44.22 (36.32-52.57) | -3.59 (-3.7--3.49) |
| Commonwealth Low Income | 96516 (78374-118623) | 116.26 (93.74-142.61) | 142944 (106722-181732) | 67.74 (50.58-85.3) | -1.88 (-2.03--1.74) |
| Commonwealth Middle Income | 651693 (522580-793262) | 112.47 (88.55-137.81) | 1024861 (814454-1248795) | 71.69 (56.76-87.5) | -1.4 (-1.5--1.3) |
| East Asia | 1497417 (1233278-1799582) | 212.71 (175.07-254.13) | 2360632 (1870733-2957489) | 115.03 (90.85-142.91) | -2.09 (-2.19--1.99) |
| East Asia & Pacific - WB | 293453 (223737-377699) | 29.18 (21.68-37.78) | 3085379 (2492125-3736174) | 95.43 (77.08-115.34) | -0.94 (-2.07-0.2) |
| Eastern Africa | 47516 (36976-59739) | 70.74 (55.77-89.01) | 56144 (44447-69722) | 36.18 (28.65-44.88) | -2.46 (-2.56--2.35) |
| Eastern Europe | 329887 (292168-369358) | 118.19 (104.47-132.73) | 287407 (240325-336244) | 83.22 (69.67-97.3) | -1.65 (-2.19--1.11) |
| Eastern Mediterranean Region | 183369 (150764-217121) | 108.42 (88.37-129.21) | 311058 (251290-371147) | 73.06 (58.71-87.45) | -1.41 (-1.54--1.28) |
| Eastern Sub-Saharan Africa | 48452 (37903-61545) | 67.28 (52.53-86.07) | 58755 (46123-73035) | 35.22 (27.74-44.06) | -2.34 (-2.43--2.24) |
| Europe | 1221561 (1063182-1387286) | 118.93 (103.55-134.93) | 959851 (801865-1118462) | 61.24 (51.5-70.91) | -2.36 (-2.53--2.19) |
| Europe & Central Asia - WB | 1249142 (1086841-1418316) | 118.32 (102.96-134.26) | 990652 (828212-1152308) | 61.14 (51.42-70.78) | -2.35 (-2.53--2.17) |
| European Region | 1256080 (1092752-1426353) | 118.19 (102.83-134.12) | 997931 (834188-1160188) | 60.96 (51.25-70.54) | -2.36 (-2.54--2.18) |
| High-income Asia Pacific | 162144 (137253-187842) | 83.47 (70.32-97.16) | 175295 (143444-208657) | 34.42 (28.36-40.34) | -3.01 (-3.06--2.97) |
| High-income North America | 406600 (345961-469740) | 115.92 (98.89-133.46) | 374913 (307110-446726) | 55.97 (46.08-66.52) | -2.52 (-2.59--2.44) |
| Latin America & Caribbean - WB | 219391 (185286-253636) | 84.81 (71.14-98.71) | 258011 (209357-307083) | 36.74 (29.75-43.78) | -2.84 (-2.92--2.75) |
| Limited Health System | 860919 (691411-1036536) | 117.66 (93.83-142.26) | 1300184 (1038124-1563013) | 72.3 (57.09-87.36) | -1.56 (-1.63--1.49) |
| Middle East & North Africa - WB | 108448 (90799-127019) | 93.79 (77.95-110.87) | 209374 (165347-251283) | 64.22 (50.28-77.53) | -1.23 (-1.25--1.2) |
| Minimal Health System | 30994 (23541-39187) | 52.09 (39.86-66.03) | 45077 (33780-56427) | 45077 (33780-56427) | -1.23 (-1.26--1.21) |
| North Africa and Middle East | 172056 (144073-198971) | 107.22 (89.19-124.79) | 293550 (236496-352908) | 68.15 (54.7-82.25) | -1.53 (-1.59--1.46) |
| North America | 406548 (345912-469680) | 115.9 (98.87-133.43) | 374872 (307074-446685) | 55.96 (46.07-66.5) | -2.52 (-2.59--2.44) |
| Northern Africa | 55665 (46321-65431) | 97.19 (80.09-115.26) | 106411 (82587-131807) | 74.78 (57.74-92.99) | -0.76 (-0.82--0.7) |
| Oceania | 3868 (2897-4950) | 129.81 (97.75-167.37) | 7416 (5620-9479) | 94.3 (71.21-121.31) | -1.12 (-1.17--1.07) |
| Region of the Americas | 623986 (531032-721635) | 103.06 (87.63-119.23) | 631069 (518622-754412) | 46.42 (38.2-55.43) | -2.74 (-2.81--2.67) |
| South-East Asia Region | 821667 (662926-982833) | 127.16 (101.43-153.22) | 1336112 (1080680-1595581) | 79.79 (64.45-95.51) | -1.46 (-1.53--1.39) |
| South Asia | 677579 (545275-824816) | 128.3 (101.23-157.12) | 1065686 (843423-1295684) | 79.03 (62.32-96.09) | -1.51 (-1.6--1.43) |
| South Asia - WB | 691174 (556606-840308) | 127 (100.4-155.36) | 1081135 (855040-1314168) | 78.14 (61.6-94.96) | -1.51 (-1.6--1.43) |
| Southeast Asia | 303228 (249394-360003) | 127.44 (104-152.1) | 535682 (440761-635245) | 85.92 (70.99-101.27) | -1.36 (-1.45--1.26) |
| Southern Africa | 39479 (31582-48070) | 91.87 (73.27-112.86) | 56899 (45505-69419) | 58.89 (46.86-71.45) | -1.51 (-1.8--1.22) |
| Southern Latin America | 38875 (33180-44783) | 83.72 (71.32-96.92) | 35435 (29141-41907) | 40.91 (33.76-48.33) | -2.09 (-2.19--1.99) |
| Southern Sub-Saharan Africa | 27756 (22402-33601) | 101.97 (81.85-124.13) | 36725 (29334-44539) | 62.24 (49.81-75.96) | -1.65 (-2.02--1.28) |
| Sub-Saharan Africa - WB | 123998 (97959-154049) | 57.17 (45.22-70.55) | 167143 (131197-204167) | 33.43 (26.39-41.01) | -1.88 (-1.99--1.76) |
| Tropical Latin America | 106918 (89846-123343) | 122.04 (101.55-143.21) | 120787 (97526-146169) | 47.21 (38.07-57.18) | -3.23 (-3.34--3.13) |
| Western Africa | 22203 (16649-28307) | 28.52 (21.63-36.87) | 32534 (24075-40969) | 18.5 (13.82-23.42) | -1.44 (-1.56--1.33) |
| Western Europe | 616260 (525822-707513) | 106.1 (90.85-121.48) | 425437 (351408-501667) | 44.28 (36.87-51.75) | -2.83 (-2.89--2.77) |
| Western Pacific Region | 1754552 (1469598-2083156) | 173.67 (144.63-206.29) | 2700611 (2170774-3319011) | 96.38 (77.39-118.02) | -2 (-2.07--1.94) |
| Western Sub-Saharan Africa | 25640 (19281-32433) | 29.72 (22.47-37.83) | 38296 (28258-48298) | 19.41 (14.44-24.59) | -1.42 (-1.56--1.29) |
| World Bank High Income | 1413186 (1211682-1621565) | 109.7 (94.12-125.78) | 1168393 (967098-1374415) | 48.46 (40.38-56.56) | -2.73 (-2.78--2.69) |
| World Bank Low Income | 98467 (77664-120450) | 70.48 (55.6-85.81) | 141565 (109663-173211) | 44.81 (34.71-55) | -1.59 (-1.64--1.55) |
| World Bank Lower Middle Income | 1156668 (955018-1369884) | 118.77 (97.41-141.2) | 1821720 (1482901-2157507) | 78.53 (63.75-93.29) | -1.36 (-1.44--1.27) |
| World Bank Upper Middle Income | 2110699 (1780006-2459418) | 154.5 (130.12-180.3) | 3038232 (2450608-3688374) | 89.36 (71.88-108.35) | -1.96 (-2.09--1.83) |
| **Country** |  |  |  |  |  |
| Afghanistan | 4904 (3387-6703) | 73.08 (51.77-99.03) | 6286 (4457-8507) | 60.68 (43.6-80.14) | -0.63 (-0.99--0.27) |
| Albania | 2524 (2093-2941) | 137.51 (113.46-161.19) | 4057 (3232-5017) | 94.68 (75.28-117.38) | -0.95 (-1.21--0.7) |
| Algeria | 9762 (7681-12063) | 109.53 (85.68-137.18) | 16778 (12524-22125) | 61.5 (45.48-80.98) | -1.86 (-2.02--1.7) |
| American Samoa | 25 (20-31) | 113.34 (89.5-136.63) | 43 (34-54) | 88.46 (69.72-111.5) | -0.92 (-1--0.83) |
| Andorra | 51 (38-70) | 91.52 (67.32-123.99) | 67 (45-91) | 42.01 (28.11-57.24) | -2.33 (-2.6--2.06) |
| Angola | 3714 (2699-4835) | 91.09 (66.59-118.28) | 6883 (4878-9093) | 56.34 (40.54-74.59) | -1.61 (-1.8--1.43) |
| Antigua and Barbuda | 21 (16-25) | 38.55 (30.02-46.97) | 26 (20-32) | 24.68 (19.35-30.64) | -1.69 (-1.93--1.46) |
| Argentina | 29238 (24759-33666) | 90.2 (76.44-104.27) | 26477 (21579-31555) | 47.67 (38.91-56.75) | -1.78 (-1.9--1.66) |
| Armenia | 3656 (3214-4088) | 135.7 (118.12-152.71) | 4192 (3566-4893) | 96.23 (81.86-112.46) | -1.32 (-1.48--1.16) |
| Australia | 15837 (13257-18501) | 80 (66.96-93.58) | 11916 (9470-14679) | 25.99 (20.88-31.76) | -3.68 (-3.78--3.58) |
| Austria | 8820 (7447-10303) | 75.99 (64.44-87.97) | 7898 (6400-9562) | 43.41 (35.63-51.56) | -1.77 (-1.86--1.68) |
| Azerbaijan | 5873 (4893-6811) | 115.45 (95.88-134.31) | 8658 (7062-10237) | 89.98 (73.68-107.5) | -0.63 (-0.91--0.35) |
| Bahamas | 68 (54-84) | 43.37 (34-53.91) | 117 (83-153) | 28.79 (20.22-37.26) | -1.22 (-1.31--1.12) |
| Bahrain | 232 (187-281) | 158.65 (125.86-195.68) | 469 (372-592) | 69.38 (53.91-90.02) | -3.26 (-3.58--2.94) |
| Bangladesh | 69787 (56556-85814) | 154.45 (124.58-189.44) | 104021 (77593-133520) | 81.22 (60.99-103.13) | -2.14 (-2.34--1.94) |
| Barbados | 103 (80-128) | 34.59 (27.14-42.74) | 104 (73-139) | 20.12 (14.09-26.88) | -2.18 (-2.42--1.93) |
| Belarus | 16826 (14505-19170) | 130.73 (112.41-149.47) | 17308 (13395-21324) | 108.36 (84.22-133.24) | -1.16 (-1.51--0.82) |
| Belgium | 19085 (16130-22052) | 123.38 (104.72-142.07) | 11873 (9588-14241) | 49.61 (40.97-58.65) | -2.9 (-2.99--2.81) |
| Belize | 38 (31-45) | 41.08 (33.44-48.92) | 90 (70-113) | 31.04 (23.93-39.19) | -1.14 (-1.55--0.74) |
| Benin | 753 (557-987) | 37.92 (27.87-49.65) | 877 (611-1183) | 16.98 (11.84-22.84) | -2.74 (-2.88--2.61) |
| Bermuda | 45 (36-55) | 72.55 (57.69-88.83) | 45 (34-58) | 32.24 (24.85-41.63) | -2.45 (-2.65--2.24) |
| Bhutan | 140 (94-206) | 66.16 (44.22-97.24) | 251 (172-350) | 44.62 (30.7-62.3) | -1.32 (-1.43--1.2) |
| Bolivia (Plurinational State of) | 1882 (1421-2418) | 60.15 (45.23-77.56) | 2521 (1861-3585) | 29.13 (21.76-41.09) | -1.97 (-2.27--1.67) |
| Bosnia and Herzegovina | 4886 (4191-5598) | 122.16 (103.71-141.59) | 5829 (4455-7291) | 92.63 (70.59-115.96) | -0.97 (-1.11--0.83) |
| Botswana | 787 (568-1072) | 143.82 (104.38-194.05) | 985 (717-1333) | 68.96 (51.53-92.3) | -2.62 (-2.9--2.34) |
| Brazil | 105147 (88335-121278) | 122.91 (101.99-144.3) | 117324 (94510-142015) | 46.89 (37.74-56.89) | -3.29 (-3.39--3.18) |
| Brunei Darussalam | 148 (119-177) | 154.24 (123.86-186.54) | 186 (144-231) | 58.49 (44.6-72.52) | -2.72 (-3--2.45) |
| Bulgaria | 17740 (15010-20369) | 149.12 (126.93-171.45) | 11889 (9556-14411) | 91.26 (73.33-110.16) | -1.9 (-2.08--1.73) |
| Burkina Faso | 1116 (800-1507) | 24.89 (17.97-33.27) | 1774 (1251-2406) | 17.91 (12.79-24.16) | -0.97 (-1.15--0.78) |
| Burundi | 2199 (1648-2864) | 92.69 (69.84-120.6) | 1624 (1172-2136) | 31.69 (22.71-41.59) | -3.75 (-4.24--3.27) |
| Cabo Verde | 66 (49-86) | 29.72 (21.98-38.66) | 99 (73-132) | 21.96 (16.21-28.88) | -1.51 (-1.97--1.05) |
| Cambodia | 7762 (6274-9426) | 186.36 (150.24-225.41) | 14428 (10747-17918) | 131.07 (97.42-163.04) | -1.32 (-1.47--1.17) |
| Cameroon | 1752 (1282-2298) | 37.98 (27.85-49.31) | 3267 (2085-4588) | 24.59 (16.04-34.49) | -1.41 (-1.75--1.06) |
| Canada | 36914 (31600-42677) | 113.57 (97.27-131.49) | 32295 (26183-38537) | 42.73 (35.08-50.71) | -3.23 (-3.31--3.15) |
| Central African Republic | 1200 (879-1608) | 96.14 (70.94-126.66) | 1549 (1011-2168) | 59.93 (41.46-81.82) | -1.66 (-1.82--1.5) |
| Chad | 1218 (851-1673) | 44.02 (30.69-60.57) | 2002 (1324-2836) | 35.36 (23.55-51.04) | -0.97 (-1.21--0.74) |
| Chile | 5704 (4748-6794) | 55.96 (46.44-66.88) | 5524 (4485-6611) | 21.76 (17.74-26.03) | -2.9 (-2.95--2.84) |
| China | 1462999 (1200179-1760241) | 217.27 (178.48-260.26) | 2304209 (1814317-2896305) | 116.69 (91.86-145.56) | -2.11 (-2.21--2.01) |
| Colombia | 9188 (7654-10967) | 53.21 (44.15-63.56) | 10772 (8076-14270) | 19.35 (14.53-25.54) | -3.79 (-3.97--3.61) |
| Comoros | 141 (95-193) | 77.25 (53.79-103.97) | 176 (123-237) | 39.44 (27.79-52.91) | -2.55 (-2.79--2.32) |
| Congo | 747 (557-990) | 69.26 (52.19-90.97) | 1318 (952-1747) | 49.67 (36.9-64.8) | -1.15 (-1.4--0.91) |
| Cook Islands | 13 (10-16) | 106.38 (81.5-130.71) | 15 (12-20) | 60.3 (46.29-77.56) | -1.96 (-2.07--1.84) |
| Costa Rica | 904 (732-1081) | 53.55 (43.1-64.35) | 1371 (1045-1721) | 24.79 (18.94-31.1) | -2.73 (-2.95--2.52) |
| Croatia | 9454 (8023-10962) | 162.56 (136.73-190.53) | 7538 (6101-9023) | 82.13 (66.84-97.87) | -1.99 (-2.08--1.9) |
| Cuba | 10550 (8890-12319) | 104.98 (88.28-123.12) | 13319 (10615-16157) | 67.19 (53.66-81.2) | -1.57 (-1.68--1.46) |
| Cyprus | 791 (652-929) | 121.45 (98.56-146.37) | 1065 (854-1293) | 52.28 (41.89-63.77) | -2.72 (-2.8--2.64) |
| Czechia | 21364 (18086-24904) | 155.3 (131.61-181.07) | 13823 (11187-16684) | 63.56 (51.44-76.76) | -2.8 (-2.88--2.73) |
| C?te d'Ivoire | 2128 (1536-2821) | 52.11 (38.06-69.19) | 3581 (2458-4961) | 31.15 (21.62-42.32) | -2.25 (-2.64--1.86) |
| Democratic People's Republic of Korea | 20297 (15038-25890) | 136.23 (101.4-171.51) | 34352 (25487-42926) | 105.52 (78.92-132.42) | -0.9 (-1--0.79) |
| Democratic Republic of the Congo | 6928 (5083-9102) | 42.73 (31.53-55.84) | 11143 (7855-15233) | 27.5 (19.58-37.17) | -1.38 (-1.54--1.22) |
| Denmark | 14877 (12903-17021) | 180.28 (156.69-205.07) | 9076 (7376-10779) | 71 (58.01-83.77) | -3.27 (-3.38--3.16) |
| Djibouti | 122 (86-174) | 91.33 (65.28-126.38) | 393 (264-549) | 66.21 (45.43-90.98) | -1.1 (-1.17--1.02) |
| Dominica | 25 (20-31) | 42.86 (34.06-52.07) | 26 (20-34) | 31.18 (23.77-40.97) | -1.02 (-1.28--0.76) |
| Dominican Republic | 2431 (1882-3055) | 73.17 (56.62-92.81) | 5730 (4313-7615) | 58.16 (43.74-77.51) | -0.28 (-0.54--0.02) |
| Ecuador | 2406 (1946-2954) | 47.52 (38.33-58.7) | 3028 (2194-3957) | 19.15 (13.87-25.09) | -2.69 (-2.88--2.5) |
| Egypt | 29406 (24336-34805) | 120.51 (97.75-145.42) | 61816 (48174-77149) | 110.51 (84.97-137.97) | 0.03 (-0.16-0.21) |
| El Salvador | 724 (556-913) | 24.1 (18.42-30.58) | 1225 (886-1615) | 19.84 (14.34-26.14) | -0.66 (-0.85--0.46) |
| Equatorial Guinea | 155 (108-213) | 75.7 (52.82-103.83) | 175 (112-246) | 33.6 (22.04-46.19) | -2.92 (-3.47--2.36) |
| Eritrea | 885 (594-1267) | 59.7 (40.89-86.53) | 1051 (688-1504) | 30.78 (20.4-43.74) | -2.47 (-2.6--2.33) |
| Estonia | 2385 (2053-2742) | 116.83 (100.37-134.27) | 1203 (979-1426) | 46.81 (38.33-55.25) | -3.47 (-3.81--3.12) |
| Eswatini | 206 (152-274) | 77.22 (57.13-104.71) | 284 (192-393) | 52.07 (35.56-71.2) | -0.91 (-1.44--0.37) |
| Ethiopia | 9682 (7299-12601) | 44.51 (33.67-58.4) | 5933 (4250-7959) | 13.75 (9.77-18.51) | -3.8 (-4.12--3.48) |
| Fiji | 525 (404-655) | 136.13 (104.38-170.72) | 716 (516-972) | 87.92 (64.57-119.07) | -1.66 (-1.8--1.52) |
| Finland | 5507 (4621-6481) | 78.24 (65.98-91.7) | 3854 (3093-4679) | 30.46 (24.63-36.53) | -2.95 (-2.99--2.9) |
| France | 64926 (54969-75741) | 79 (67.31-91.51) | 52332 (42227-62096) | 38.06 (31.43-44.26) | -2.25 (-2.3--2.2) |
| Gabon | 257 (190-332) | 44.75 (32.83-57.87) | 390 (267-519) | 35.92 (24.88-47.27) | -0.72 (-0.79--0.65) |
| Gambia | 246 (172-328) | 67.12 (47.39-89.22) | 377 (257-512) | 37.21 (25.54-49.82) | -2.14 (-2.31--1.98) |
| Georgia | 7015 (5994-7965) | 111.84 (95.78-127.56) | 5342 (4501-6293) | 91.12 (76.93-106.85) | -0.46 (-0.65--0.27) |
| Germany | 132907 (111777-155709) | 104.36 (88.01-121.84) | 94196 (76715-112178) | 48.57 (40.28-57.28) | -2.41 (-2.57--2.24) |
| Ghana | 2012 (1487-2710) | 34.83 (25.69-46.03) | 4542 (3231-6103) | 29.38 (20.59-39.01) | 0.09 (-0.11-0.28) |
| Greece | 17912 (15559-20534) | 119.55 (103.42-137.08) | 18251 (15385-21438) | 74.44 (63.81-85.41) | -1.54 (-1.62--1.46) |
| Greenland | 87 (73-101) | 265.76 (222.4-314.54) | 80 (65-98) | 115.88 (92.39-141.98) | -2.69 (-2.76--2.61) |
| Grenada | 33 (26-41) | 47.53 (38.16-59.44) | 32 (24-40) | 27.38 (20.78-34.51) | -2.06 (-2.35--1.78) |
| Guam | 55 (45-66) | 68.47 (55.59-82.46) | 106 (84-131) | 50.81 (40.68-62.77) | -0.56 (-0.78--0.34) |
| Guatemala | 1447 (1118-1837) | 47.6 (36.17-61.29) | 2223 (1629-2909) | 21.26 (15.46-27.81) | -2.97 (-3.15--2.8) |
| Guinea | 1332 (959-1810) | 41.62 (29.94-56.54) | 1996 (1364-2722) | 35.96 (24.73-49.56) | -0.28 (-0.45--0.11) |
| Guinea-Bissau | 166 (115-226) | 39.29 (27.08-53.1) | 266 (186-365) | 32.48 (23.06-44.19) | -0.11 (-0.37-0.14) |
| Guyana | 269 (215-338) | 67.88 (53.8-84.92) | 251 (174-348) | 37.97 (26.62-52.3) | -1.52 (-1.62--1.42) |
| Haiti | 1959 (1489-2533) | 57.8 (43.97-73.35) | 2271 (1541-3168) | 31.16 (21.24-42.67) | -1.88 (-2.08--1.69) |
| Honduras | 1062 (823-1326) | 53.94 (41.39-67.41) | 3344 (2599-4243) | 57.08 (43.85-71.82) | 0.52 (0.33-0.72) |
| Hungary | 22327 (19363-25607) | 154.84 (134.32-176.86) | 15320 (12616-18392) | 82.44 (68.23-98.35) | -2.15 (-2.25--2.06) |
| Iceland | 298 (245-360) | 102.61 (84.98-122.87) | 244 (192-305) | 40.42 (32.09-49.71) | -3.07 (-3.14--2.99) |
| India | 515814 (408670-633963) | 121.88 (93.88-150.92) | 836057 (657238-1028421) | 76.76 (60.13-94.92) | -1.38 (-1.5--1.25) |
| Indonesia | 92941 (73966-113283) | 100.28 (78.68-123.35) | 223481 (169965-283927) | 102.8 (78.25-129.53) | 0.18 (0-0.36) |
| Iran (Islamic Republic of) | 15612 (12679-18595) | 61.19 (49.08-73.73) | 28860 (23745-34397) | 38.12 (30.8-45.71) | -1.34 (-1.43--1.25) |
| Iraq | 10433 (8319-12835) | 134.96 (107.05-166.23) | 21408 (15414-27370) | 100.4 (73.44-126.63) | -1.48 (-1.66--1.31) |
| Ireland | 7202 (6080-8354) | 177.05 (148.86-205.84) | 3617 (2873-4360) | 44.01 (35.19-52.76) | -4.59 (-4.76--4.41) |
| Israel | 3883 (3224-4622) | 82.25 (68.05-97.54) | 3698 (3001-4449) | 29.26 (23.85-34.97) | -3.42 (-3.49--3.36) |
| Italy | 82620 (70061-94904) | 93.15 (79.17-106.95) | 56648 (45722-67391) | 36.95 (30.49-43.21) | -3.02 (-3.06--2.97) |
| Jamaica | 835 (676-1000) | 47.17 (38.21-56.31) | 1054 (745-1454) | 33.96 (24.09-46.76) | -1.22 (-1.58--0.85) |
| Japan | 126240 (107611-146855) | 76.13 (64.72-88.71) | 132467 (107263-158034) | 33.55 (27.85-39.25) | -2.77 (-2.81--2.73) |
| Jordan | 1335 (1077-1638) | 108.76 (86.22-134.18) | 3572 (2730-4474) | 52.67 (40.25-65.51) | -2.68 (-2.85--2.5) |
| Kazakhstan | 15596 (13390-17992) | 118.62 (101.33-137.86) | 12415 (10169-14889) | 68.16 (55.5-81.45) | -2.47 (-2.95--2) |
| Kenya | 4197 (2964-5780) | 53.67 (38.13-74.85) | 7721 (5624-10379) | 34.33 (24.8-46.12) | -1.6 (-1.8--1.39) |
| Kiribati | 92 (73-114) | 243.82 (192.5-300.06) | 167 (127-213) | 223.43 (169.83-282.69) | -0.42 (-0.62--0.22) |
| Kuwait | 439 (363-519) | 69.17 (55.51-82.7) | 1262 (953-1605) | 42.35 (31.3-54.73) | -1.39 (-1.82--0.95) |
| Kyrgyzstan | 3727 (3132-4336) | 127.41 (107.25-148.61) | 4265 (3391-5255) | 92.2 (74.72-113.36) | -0.81 (-1.14--0.49) |
| Lao People's Democratic Republic | 4120 (3093-5165) | 206.85 (156.9-257.34) | 5281 (3882-6666) | 126.63 (94.34-158.78) | -1.66 (-1.72--1.61) |
| Latvia | 4299 (3710-4926) | 120.96 (104.22-138.38) | 2343 (1904-2786) | 63.79 (52.22-75.43) | -2.48 (-2.77--2.19) |
| Lebanon | 2787 (2176-3489) | 134.38 (105.4-168.61) | 4893 (3821-6084) | 77.95 (61.08-96.66) | -1.47 (-1.62--1.33) |
| Lesotho | 874 (629-1171) | 106.76 (77.32-144.04) | 1814 (1323-2354) | 164.14 (118.11-214.68) | 2.21 (1.8-2.62) |
| Liberia | 356 (252-471) | 31.35 (22.42-41.25) | 477 (314-667) | 20.45 (13.53-28.19) | -1.65 (-1.8--1.5) |
| Libya | 1321 (1038-1675) | 72.01 (56.43-91.65) | 3663 (2728-4759) | 70.82 (52.68-91.34) | 0.4 (0.21-0.6) |
| Lithuania | 5146 (4375-5930) | 114.99 (97.95-132.58) | 3578 (2930-4272) | 64.95 (53.69-77.02) | -1.96 (-2.22--1.7) |
| Luxembourg | 572 (471-682) | 105.33 (86.84-125.42) | 454 (357-564) | 42.1 (33.35-52.06) | -2.93 (-3--2.86) |
| Madagascar | 3789 (2971-4780) | 78.1 (60.53-100.56) | 3514 (2376-5024) | 31.88 (21.81-44.72) | -3.08 (-3.34--2.83) |
| Malawi | 2604 (1958-3324) | 75.34 (56.4-97.19) | 4483 (3221-5890) | 62.47 (45.45-81.36) | -0.88 (-1.16--0.61) |
| Malaysia | 9761 (8187-11466) | 110.21 (91.62-129.94) | 19259 (15934-22759) | 71.93 (59.02-85.7) | -1.59 (-1.78--1.4) |
| Maldives | 128 (106-152) | 176.69 (145.91-211.13) | 166 (129-208) | 55.96 (43.68-69.71) | -4.08 (-4.25--3.91) |
| Mali | 1199 (832-1585) | 33.31 (23.05-44.06) | 2743 (1917-3708) | 35.68 (24.63-47.78) | 0.67 (0.5-0.84) |
| Malta | 403 (334-475) | 94.82 (78.55-112.79) | 317 (254-387) | 33.72 (27.1-40.78) | -3.38 (-3.46--3.31) |
| Marshall Islands | 24 (19-30) | 139.99 (107.04-177.66) | 46 (33-61) | 121.18 (87.97-160.41) | -0.31 (-0.4--0.22) |
| Mauritania | 452 (327-594) | 43.84 (31.75-57.91) | 467 (311-630) | 21.43 (14.44-28.79) | -2.47 (-2.73--2.22) |
| Mauritius | 806 (673-946) | 112.99 (93.42-134.38) | 991 (825-1164) | 55.01 (45.79-64.7) | -2.51 (-2.81--2.21) |
| Mexico | 26154 (21386-31011) | 71.37 (57.68-85.38) | 32299 (25184-40098) | 26.64 (20.68-33.08) | -3.47 (-3.63--3.32) |
| Micronesia (Federated States of) | 94 (72-121) | 188.03 (145.04-241.56) | 119 (88-158) | 148.62 (110.29-193.11) | -0.79 (-0.84--0.75) |
| Monaco | 68 (51-86) | 98.48 (73.51-123.87) | 71 (57-89) | 73.19 (58.44-92.96) | -0.89 (-1.02--0.76) |
| Mongolia | 1361 (1092-1650) | 129.43 (104.1-156.55) | 2368 (1851-2919) | 99.48 (76.06-122.36) | -1 (-1.22--0.78) |
| Montenegro | 771 (660-885) | 122.66 (104.48-141.83) | 1120 (918-1342) | 114.39 (93.48-137.17) | -0.23 (-0.47-0) |
| Morocco | 9997 (8093-12102) | 70.22 (56.28-85.49) | 14559 (10783-18055) | 42.14 (31.36-52.81) | -1.73 (-1.91--1.54) |
| Mozambique | 3412 (2474-4515) | 59.18 (42.8-77.4) | 5938 (4386-7940) | 51.56 (37.41-68.9) | 0.18 (-0.03-0.39) |
| Myanmar | 57952 (44299-72512) | 277.38 (213.78-348.16) | 47058 (35380-60207) | 108.83 (81.43-139.73) | -3.35 (-3.48--3.21) |
| Namibia | 680 (506-881) | 123.77 (91.2-159.77) | 875 (631-1135) | 73.06 (53.52-94.24) | -1.98 (-2.33--1.62) |
| Nauru | 12 (9-15) | 242.21 (183.12-307.62) | 12 (9-16) | 191.69 (140.9-246.99) | -0.9 (-1.18--0.61) |
| Nepal | 18413 (13746-24516) | 226.11 (168.33-295.64) | 24097 (17656-31058) | 120.53 (89.06-155.19) | -2.11 (-2.3--1.92) |
| Netherlands | 25732 (22213-29476) | 127.71 (110.4-145.93) | 20612 (16879-24816) | 55.16 (45.41-65.66) | -2.75 (-2.83--2.67) |
| New Zealand | 3990 (3326-4670) | 101.2 (84.47-118.51) | 3400 (2757-4123) | 39.01 (31.9-47.05) | -3.2 (-3.36--3.04) |
| Nicaragua | 473 (375-584) | 31.73 (24.98-39.44) | 973 (725-1256) | 20.67 (15.3-26.54) | -1.01 (-1.21--0.81) |
| Niger | 608 (425-838) | 23.3 (16.36-31.62) | 1122 (756-1544) | 16.23 (10.96-22.16) | -1.19 (-1.25--1.12) |
| Nigeria | 8720 (6197-11947) | 20.66 (14.66-28.09) | 9684 (6571-13430) | 10.58 (7.41-14.6) | -2.36 (-2.58--2.13) |
| Niue | 2 (2-3) | 100.74 (77.11-129.22) | 2 (1-3) | 86.99 (66.12-116.51) | -0.7 (-0.79--0.61) |
| North Macedonia | 2739 (2286-3175) | 148.99 (123.36-174.74) | 3345 (2637-4120) | 108.96 (84.83-134.22) | -1.22 (-1.57--0.87) |
| Northern Mariana Islands | 21 (16-27) | 116.36 (91.16-147.15) | 43 (35-52) | 81.41 (65.68-98.98) | -1.16 (-1.23--1.08) |
| Norway | 6529 (5365-7745) | 94.81 (78.8-111.48) | 3508 (2768-4339) | 33.15 (26.67-40.5) | -3.71 (-3.88--3.53) |
| Oman | 430 (311-577) | 62.26 (44.2-84.22) | 555 (410-729) | 28.81 (21.14-37.88) | -2.02 (-2.19--1.85) |
| Pakistan | 73425 (56825-90467) | 139.7 (105.55-173.15) | 101260 (74280-134473) | 91.07 (68.15-120.27) | -1.68 (-1.97--1.39) |
| Palau | 12 (9-16) | 123.54 (94.18-162.44) | 21 (16-28) | 94.6 (69.37-125.07) | -0.87 (-0.92--0.82) |
| Palestine | 970 (739-1223) | 123.25 (94.77-154.71) | 1681 (1338-2065) | 73.85 (58.42-91.72) | -1.9 (-2.16--1.64) |
| Panama | 612 (489-748) | 43 (34.16-52.84) | 844 (608-1134) | 18.89 (13.6-25.3) | -2.95 (-3.09--2.8) |
| Papua New Guinea | 2204 (1530-2977) | 121.34 (84.01-164.65) | 4800 (3403-6504) | 90.56 (63.68-122.92) | -1.02 (-1.07--0.97) |
| Paraguay | 1770 (1433-2159) | 84.13 (67.85-102.44) | 3462 (2468-4582) | 61.94 (44.36-81.72) | -0.9 (-1--0.8) |
| Peru | 3295 (2539-4128) | 28.07 (21.65-35.28) | 5758 (4161-8019) | 17.13 (12.39-23.84) | -1.99 (-2.34--1.63) |
| Philippines | 37379 (30706-44766) | 141.43 (113.05-172.33) | 72993 (56155-91601) | 90.94 (69.88-113.56) | -1.28 (-1.43--1.12) |
| Poland | 70385 (61461-79476) | 161.72 (141.09-183.56) | 51198 (43033-60698) | 71.26 (60.06-84.27) | -2.74 (-2.8--2.67) |
| Portugal | 10104 (8401-11806) | 73.32 (60.85-85.72) | 6997 (5649-8433) | 30.53 (25.18-36.04) | -2.96 (-3.07--2.86) |
| Puerto Rico | 1869 (1456-2360) | 52.66 (40.96-66.79) | 1727 (1246-2308) | 23.91 (17.65-31.85) | -2.9 (-3.05--2.76) |
| Qatar | 102 (78-131) | 96.6 (72.81-124.03) | 317 (223-425) | 33.18 (22.99-44.44) | -3.8 (-4.39--3.21) |
| Republic of Korea | 33964 (27497-39905) | 129.74 (103.36-156.35) | 40927 (33306-49499) | 43.89 (35.61-53.08) | -3.82 (-3.97--3.67) |
| Republic of Moldova | 4775 (3985-5670) | 107.82 (89.68-128.74) | 4672 (3828-5529) | 78.67 (64.56-92.82) | -1.19 (-1.49--0.9) |
| Romania | 31528 (26770-36129) | 116.79 (98.69-135.17) | 25483 (20637-30360) | 73.19 (59.4-86.76) | -2.07 (-2.29--1.85) |
| Russian Federation | 208008 (183992-231247) | 114.28 (100.82-127.54) | 196883 (163799-229032) | 83.33 (69.5-96.86) | -1.54 (-2.15--0.92) |
| Rwanda | 4129 (3089-5289) | 160.53 (118.9-205.36) | 4250 (3055-5631) | 80.4 (58.09-106.89) | -3.17 (-3.52--2.81) |
| Saint Kitts and Nevis | 17 (13-21) | 46.22 (36.22-58.09) | 16 (11-21) | 23.46 (17.1-30.61) | -2.35 (-2.49--2.21) |
| Saint Lucia | 50 (40-62) | 59.8 (47.1-74.24) | 67 (48-88) | 27.83 (20.07-36.48) | -2.9 (-3.13--2.67) |
| Saint Vincent and the Grenadines | 27 (21-33) | 37.96 (29.83-46.41) | 40 (31-51) | 28.46 (21.71-35.89) | -1.1 (-1.23--0.98) |
| Samoa | 114 (89-141) | 137.47 (107.67-170.61) | 156 (122-195) | 108.89 (84.83-134.51) | -0.9 (-0.98--0.81) |
| San Marino | 25 (20-31) | 70.76 (56.73-86.95) | 20 (13-28) | 25.28 (15.99-36.68) | -2.5 (-2.8--2.21) |
| Sao Tome and Principe | 15 (11-20) | 23.37 (16.63-31.31) | 26 (19-36) | 23.55 (17.28-30.88) | -0.12 (-0.44-0.22) |
| Saudi Arabia | 3154 (2172-4296) | 50.77 (34.91-69.43) | 10860 (7853-14201) | 43.03 (31.7-55.08) | -0.41 (-0.59--0.22) |
| Senegal | 1517 (1123-1957) | 43.71 (32.18-56.3) | 1923 (1356-2583) | 23.43 (16.44-31.48) | -2.11 (-2.26--1.96) |
| Serbia | 14938 (12598-17312) | 139.4 (116.8-162.73) | 14822 (12018-17886) | 91.04 (73.93-109.24) | -1.62 (-1.91--1.33) |
| Seychelles | 67 (55-78) | 119.1 (97.41-139.63) | 77 (63-94) | 68.88 (56.06-84.62) | -1.66 (-1.83--1.5) |
| Sierra Leone | 1097 (792-1435) | 53.25 (38.75-69.77) | 1243 (868-1682) | 31 (21.91-42) | -1.52 (-1.68--1.35) |
| Singapore | 1793 (1459-2140) | 84.41 (68.02-101.94) | 1717 (1370-2084) | 19.92 (15.93-24.23) | -4.55 (-4.61--4.49) |
| Slovakia | 7868 (6675-9061) | 132.63 (112.86-152.64) | 5970 (4807-7201) | 62.65 (50.4-75.55) | -2.29 (-2.37--2.2) |
| Slovenia | 2064 (1730-2413) | 83.4 (69.54-97.13) | 1674 (1331-2061) | 38.63 (30.74-47.47) | -2.56 (-2.66--2.45) |
| Solomon Islands | 259 (172-339) | 189.76 (132.09-242.9) | 573 (426-749) | 157.42 (117.24-203.09) | -0.46 (-0.66--0.25) |
| Somalia | 2205 (1488-3248) | 86.03 (59.82-122.53) | 3644 (2421-5229) | 53.92 (36.65-75.41) | -1.6 (-1.66--1.53) |
| South Africa | 21524 (17485-25780) | 101.34 (82.08-122.32) | 25562 (20400-30650) | 53.95 (42.89-65.13) | -2.2 (-2.56--1.84) |
| South Sudan | 1771 (1226-2587) | 70.36 (49.18-102.26) | 1818 (1195-2503) | 47.84 (32.05-66.29) | -1.49 (-1.73--1.26) |
| Spain | 52415 (44622-59824) | 96.93 (82.49-110.6) | 41161 (33692-48894) | 41.34 (34.43-48.24) | -2.76 (-2.85--2.66) |
| Sri Lanka | 8562 (6923-10473) | 93.04 (74.67-115.36) | 8998 (5527-12773) | 35.64 (22.27-49.97) | -2.8 (-2.94--2.66) |
| Sudan | 8434 (6363-10807) | 93.69 (70.17-119.57) | 11287 (7883-15978) | 60.79 (43.22-85.51) | -1.56 (-1.63--1.48) |
| Suriname | 198 (161-237) | 76.8 (62.33-92.22) | 278 (203-379) | 43 (31.14-58.94) | -1.97 (-2.22--1.73) |
| Sweden | 12510 (10152-15129) | 80.02 (65.84-95.85) | 8529 (6644-10648) | 35.87 (28.43-44.54) | -2.46 (-2.56--2.37) |
| Switzerland | 9376 (7723-11035) | 88.63 (73.93-103.94) | 7042 (5751-8532) | 35.77 (29.54-42.59) | -2.76 (-2.86--2.67) |
| Syrian Arab Republic | 6458 (5003-8128) | 126.8 (97.89-160.48) | 10688 (7562-14445) | 89.52 (64.02-119.11) | -1.48 (-1.62--1.34) |
| Taiwan (Province of China) | 14121 (11836-16254) | 96.41 (80.05-111.89) | 22071 (18129-25801) | 51.34 (42.3-59.94) | -2.07 (-2.18--1.96) |
| Tajikistan | 3059 (2462-3686) | 113.15 (90.33-136.98) | 2544 (1868-3441) | 47.21 (34.53-65.05) | -2.91 (-3.2--2.61) |
| Thailand | 37325 (29804-44477) | 115.97 (91.67-137.81) | 56851 (40886-76758) | 52.67 (38-71.06) | -3.05 (-3.22--2.89) |
| Timor-Leste | 307 (230-417) | 119.51 (92.06-159.88) | 781 (567-1017) | 100.64 (73.05-132.04) | -0.44 (-0.6--0.28) |
| Togo | 887 (645-1173) | 74.95 (54.77-99.1) | 1829 (1245-2549) | 47.17 (32.62-65.2) | -1.56 (-1.67--1.45) |
| Tokelau | 1 (1-2) | 111.75 (83.62-145.96) | 1 (1-2) | 78.67 (57.59-104.49) | -1.28 (-1.35--1.22) |
| Tonga | 72 (57-90) | 140.94 (110.26-176.74) | 87 (66-112) | 111.16 (83.58-142.69) | -0.84 (-1--0.68) |
| Trinidad and Tobago | 621 (506-740) | 75.24 (61.57-90.25) | 716 (495-968) | 36.86 (25.45-49.79) | -2.74 (-2.98--2.5) |
| Tunisia | 4728 (3914-5652) | 105.96 (87.3-127.99) | 9129 (6625-12468) | 72.24 (52.2-98.58) | -1.54 (-1.66--1.41) |
| Turkmenistan | 2281 (1949-2632) | 118.38 (100.53-137.99) | 2802 (2061-3657) | 67.92 (50.38-88.1) | -2.39 (-2.72--2.07) |
| Tuvalu | 12 (9-15) | 170.79 (131.63-218.66) | 13 (10-17) | 123.46 (96.13-156.27) | -1.01 (-1.06--0.95) |
| Türkiye | 54584 (44961-64366) | 160.77 (132.08-189.13) | 70738 (54652-90631) | 76.78 (59.08-99.03) | -2.57 (-2.85--2.29) |
| Uganda | 2868 (2087-3937) | 47.3 (34.78-64.96) | 4167 (2936-5648) | 29.03 (20.61-39.88) | -2.52 (-2.97--2.06) |
| Ukraine | 88450 (77378-100242) | 124.89 (109.01-141.72) | 61421 (41756-85110) | 81.42 (55.13-112.76) | -1.98 (-2.47--1.49) |
| United Arab Emirates | 392 (287-522) | 85.99 (63.39-114.93) | 1316 (939-1770) | 46.13 (33.05-61.85) | -0.87 (-1.38--0.35) |
| United Kingdom | 139139 (119171-159523) | 150.65 (129.03-172.09) | 73533 (59755-88461) | 53.23 (43.64-63.79) | -3.49 (-3.61--3.37) |
| United Republic of Tanzania | 8420 (6355-10955) | 80.79 (60.8-106.02) | 11120 (8089-15211) | 44.06 (32.5-59.37) | -2.44 (-2.61--2.27) |
| United States of America | 369590 (314449-427629) | 116.32 (99.3-133.98) | 342532 (279695-408002) | 57.56 (47.2-68.24) | -2.45 (-2.52--2.37) |
| United States Virgin Islands | 39 (29-52) | 46.23 (33.82-62.53) | 42 (29-58) | 23.37 (16.35-32.18) | -2.17 (-2.36--1.98) |
| Uruguay | 3931 (3339-4468) | 101.94 (86.75-115.66) | 3432 (2787-4010) | 64.06 (52.69-74.26) | -1.68 (-1.76--1.6) |
| Uzbekistan | 6060 (4874-7266) | 51.81 (41.4-62.15) | 12436 (9648-15263) | 47.16 (36.6-58.13) | -0.33 (-0.8-0.14) |
| Vanuatu | 84 (64-107) | 132.76 (101.49-168.48) | 163 (126-204) | 90.55 (69.51-112.19) | -1.47 (-1.58--1.36) |
| Venezuela (Bolivarian Republic of) | 6302 (5137-7501) | 66.19 (53.73-79.32) | 11526 (8136-15380) | 38.57 (27.25-51.44) | -2.12 (-2.3--1.94) |
| Viet Nam | 45680 (34987-58734) | 118.4 (90.79-152.97) | 84571 (65309-101772) | 88.08 (68.69-105.38) | -1.05 (-1.11--0.99) |
| Yemen | 6483 (4790-8624) | 130.93 (97.59-171.42) | 13138 (9148-17664) | 96.88 (67.55-129.3) | -1.15 (-1.24--1.07) |
| Zambia | 1992 (1520-2561) | 74.65 (56.55-96.72) | 2870 (1952-3784) | 45.14 (31.15-60.14) | -2.29 (-2.61--1.97) |
| Zimbabwe | 3686 (2806-4803) | 98.28 (74.8-129.11) | 7205 (5157-9446) | 105.78 (76.5-137.16) | 0.65 (0.14-1.17) |
