# Supplementary figures and images for: Global, Regional, and National Burden of Smoking-Related Diseases and Associations With Health Workforce Distribution, 1990–2021: Analysis From the Global Burden of Disease Study 2021
Source: Int J Public Health. 2025 Jul 2;70:1608217. doi: 10.3389/ijph.2025.1608217 (PMC12263451; doi:10.3389/ijph.2025.1608217)

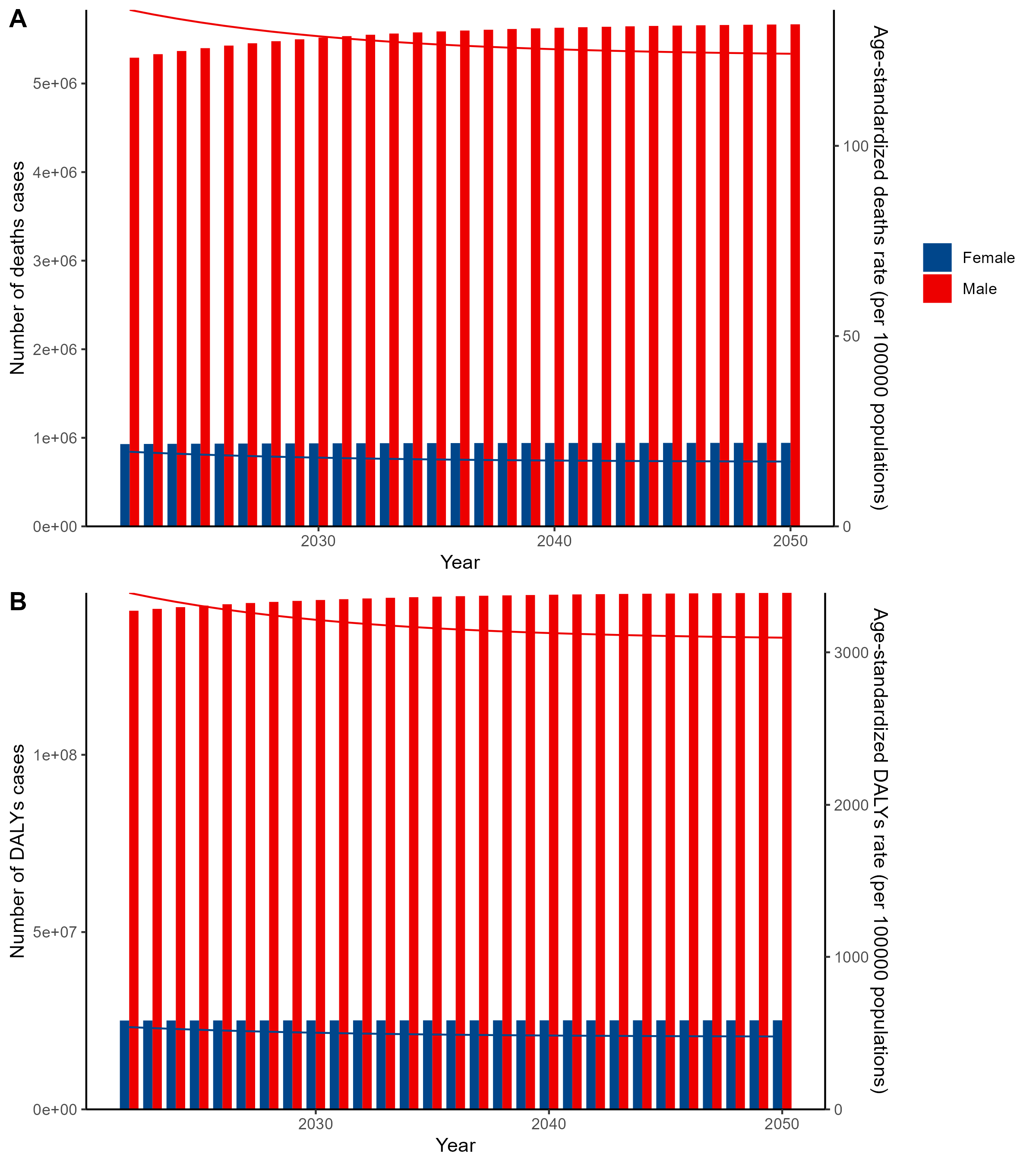

Supplement: Supplementary file 2 [file Image3.tif]

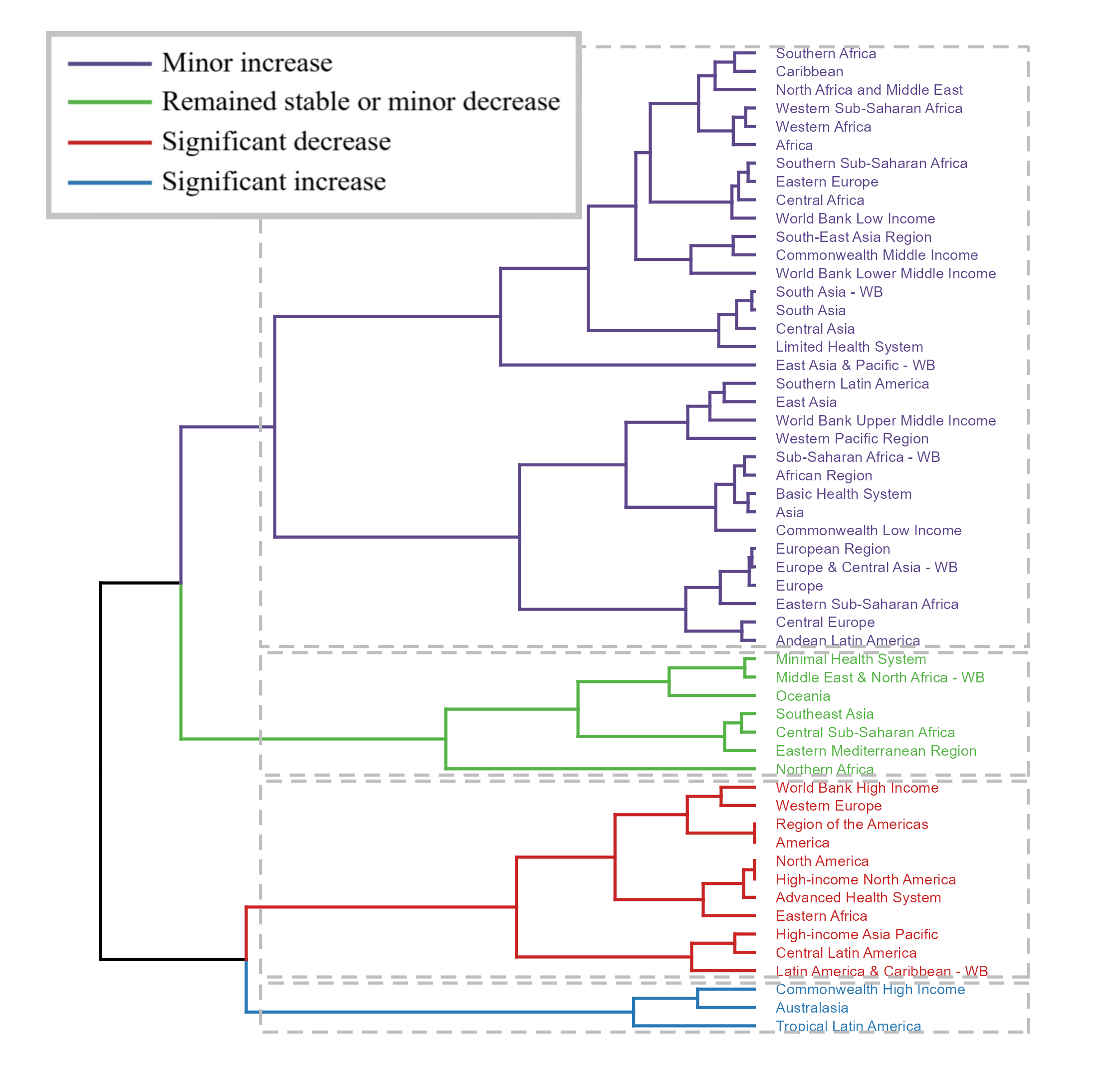

Supplement: Supplementary file 3 [file Image2.tif]

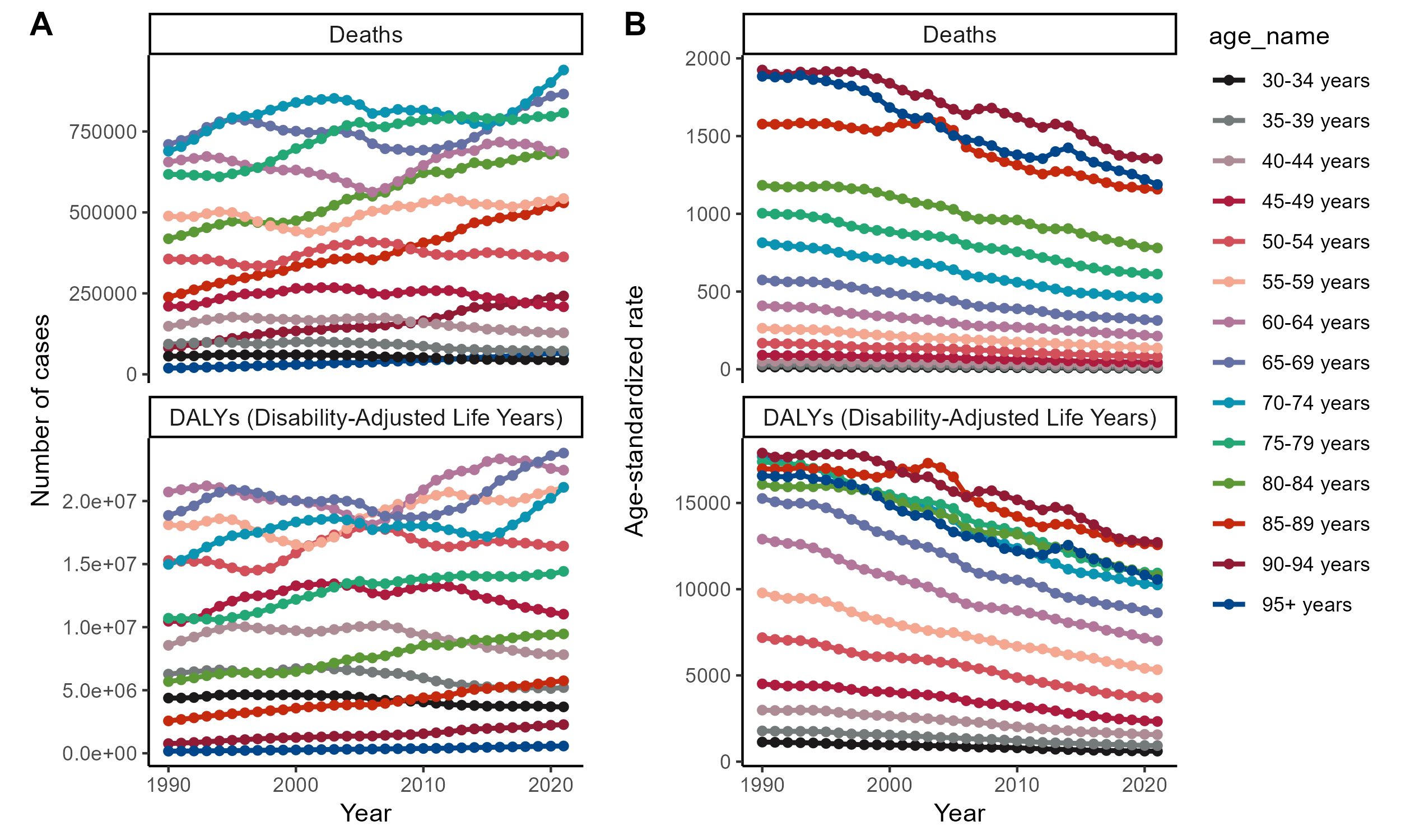

Supplement: Supplementary file 4 [file Image1.tif]
