# Supplementary material for: Global, Regional, and National Burden of Smoking-Related Diseases and Associations With Health Workforce Distribution, 1990–2021: Analysis From the Global Burden of Disease Study 2021
Source: Int J Public Health. 2025 Jul 2;70:1608217. doi: 10.3389/ijph.2025.1608217 (PMC12263451; doi:10.3389/ijph.2025.1608217)
Supplement: Supplementary file 5 [file Table2.docx]

Table S2. Number of cases of smoking DALYs and age-standardized DALYs rates in 1990 and 2021, and trends from 1990 - 2021.

| Characteristic | 1990 | | 2021 | | 1990-2021 |
| --- | --- | --- | --- | --- | --- |
|  | Number of DALYs cases (95% UI) | The age-standardized DALYs  rate/100000(95% UI) | Number of DALYs cases (95% UI) | The age-standardized DALYs  rate/100000(95% UI） | EAPC (95%CI) |
| **Global** | 137534819 (115962110-160429712) | 3360.67 (2833.61-3920.05) | 165080664 (135430162-193938448) | 1902.34 (1558.66-2234.96) | -1.95 (-2.01--1.89) |
| **Sex** |  |  |  |  |  |
| Female | 22887497  (17853711-28349206) | 1072.43 (838.61-1329.16) | 25047498 (19360377-31532703) | 547.23 (423.04-688.77) | -2.28 (-2.35--2.2) |
| Male | 114647322 (97761437-132336578) | 6002.31 (5105.29-6925.75) | 140033167 (115775585-163412336) | 3424.67 (2829.09-4001.55) | -1.92 (-1.98--1.87) |
| **Age** |  |  |  |  |  |
| <30 years | 0  (0-0) | 0 (0-0) | 0 (0-0) | 0 (0-0) | 0 (0-0) |
| 30-34 years | 4377353  (3475894-5400596) | 1135.73 (901.84-1401.21) | 3678225 (2818715-4648959) | 608.49 (466.3-769.08) | -2.11 (-2.2--2.01) |
| 35-39 years | 6275580  (5052454-7503911) | 1781.6 (1434.36-2130.31) | 5210935 (4044870-6416698) | 929.09 (721.18-1144.07) | -2.28 (-2.37--2.19) |
| 40-44 years | 8559315  (7065994-10136508) | 2987.74 (2466.47-3538.28) | 7820783 (6243909-9422500) | 1563.37 (1248.16-1883.56) | -2.34 (-2.46--2.22) |
| 45-49 years | 10469931  (8741635-12282964) | 4509.1 (3764.77-5289.92) | 11030739 (8894367-13159825) | 2329.59 (1878.41-2779.24) | -2.24 (-2.39--2.08) |
| 50-54 years | 15282637  (12999169-17780203) | 7189.41 (6115.2-8364.34) | 16427817 (13488265-19342362) | 3692.28 (3031.6-4347.35) | -2.24 (-2.35--2.12) |
| 55-59 years | 18117976  (15428826-20991360) | 9782.93 (8330.9-11334.43) | 21090308 (17358182-24699943) | 5329.5 (4386.39-6241.65) | -1.97 (-2.01--1.92) |
| 60-64 years | 20717900  (17632890-23804334) | 12899.57 (10978.75-14821.27) | 22446915 (18784020-25965798) | 7013.61 (5869.13-8113.1) | -2.01 (-2.06--1.96) |
| 65-69 years | 18862479  (16008617-21716832) | 15259.74 (12950.96-17568.91) | 23805202 (19644885-28273760) | 8630.01 (7121.78-10249.98) | -2.01 (-2.08--1.94) |
| 70-74 years | 14976646  (12597077-17192740) | 17690.1 (14879.41-20307.71) | 21086274 (17325352-25152730) | 10244.05 (8416.93-12219.6) | -1.93 (-2--1.86) |
| 75-79 years | 10706524  (8907351-12598022) | 17393.28 (14470.44-20466.11) | 14425021 (11776503-17205419) | 10937.62 (8929.41-13045.83) | -1.63 (-1.7--1.56) |
| 80-84 years | 5686685  (4613119-6837403) | 16075.01 (13040.28-19327.84) | 9460960 (7520609-11400011) | 10802.27 (8586.83-13016.23) | -1.38 (-1.47--1.29) |
| 85-89 years | 2566189  (2025236-3149839) | 16982.18 (13402.33-20844.58) | 5748022 (4523268-7025470) | 12571.73 (9893.02-15365.69) | -1.15 (-1.3--1) |
| 90-94 years | 766790  (596164-947008) | 17893.97 (13912.21-22099.57) | 2273958 (1785365-2779706) | 12711.22 (9980.03-15538.31) | -1.18 (-1.28--1.08) |
| 95+ years | 168813  (129483-211498) | 16581.44 (12718.24-20774.11) | 575508 (434246-722263) | 10559.18 (7967.36-13251.79) | -1.53 (-1.63--1.44) |
| **SDI region** |  |  |  |  |  |
| High-middle SDI | 38451659  (33013015-44305815) | 3786.88 (3241.84-4367.97) | 43940333 (36601568-52069342) | 2250.72 (1872.92-2661.29) | -1.93 (-2.11--1.75) |
| High SDI | 33056460  (27693211-38694914) | 3079 (2576.13-3611.91) | 27838145 (22484857-33301169) | 1477.18 (1184.98-1773.25) | -2.43 (-2.46--2.4) |
| Low-middle SDI | 21625863  (17528709-25917757) | 3282.33 (2638.36-3938.66) | 31485070 (25547142-37544450) | 2113.55 (1710.48-2525.23) | -1.39 (-1.43--1.35) |
| Low SDI | 5534558  (4369271-6898138) | 2236.02 (1760.79-2789.16) | 7391465 (5795582-9080169) | 1350.61 (1057.16-1664.63) | -1.73 (-1.81--1.65) |
| Middle SDI | 38702318  (32742976-45809933) | 3598.21 (3029.81-4246.91) | 54276330 (43958248-64578335) | 1993.02 (1609.87-2372.35) | -2 (-2.04--1.96) |
| **GBD region** |  |  |  |  |  |
| Advanced Health System | 51359518  (43475010-59485305) | 3225.5 (2725.74-3741.51) | 42355350 (34499289-50227364) | 1674.62 (1357.67-1997.91) | -2.31 (-2.44--2.18) |
| Africa | 5903137  (4743997-7210907) | 1859 (1492.7-2279.17) | 9152404 (7204822-11246666) | 1223.53 (956.14-1499.24) | -1.41 (-1.49--1.33) |
| African Region | 4111583  (3237354-5145279) | 1638.62 (1291.32-2033.4) | 5788873 (4476686-7131248) | 973.83 (758.47-1199.52) | -1.81 (-1.91--1.71) |
| America | 18100830  (15119000-21152042) | 2957.75 (2472.75-3451.76) | 17952194 (14379134-21814479) | 1363.45 (1088.14-1661.83) | -2.64 (-2.7--2.58) |
| Andean Latin America | 230000  (181444-280267) | 1039.7 (817.35-1268.07) | 334293 (252236-431120) | 549.17 (414.57-707.14) | -2.12 (-2.33--1.91) |
| Asia | 78717577  (65688678-92480197) | 3757.96 (3124.38-4403.92) | 111107808 (90652966-132837027) | 2171.99 (1771.5-2600.66) | -1.83 (-1.86--1.79) |
| Australasia | 562773  (452872-671221) | 2420.62 (1945.84-2889.34) | 435811 (337153-549950) | 918.59 (706.42-1161.19) | -3.16 (-3.26--3.06) |
| Basic Health System | 58186800  (49176739-68793579) | 3825.58 (3221.02-4508.76) | 84322600 (68516091-101297369) | 2229.47 (1810.03-2682.91) | -1.81 (-1.86--1.76) |
| Caribbean | 570134  (470163-672411) | 2151.26 (1774.05-2534.62) | 748642 (599755-907210) | 1390.47 (1113.32-1685.51) | -1.47 (-1.54--1.39) |
| Central Africa | 498358  (381153-639296) | 1510.18 (1151.64-1921.18) | 792650 (588114-1016557) | 939.23 (702.63-1201.3) | -1.55 (-1.61--1.49) |
| Central Asia | 1560748  (1337568-1784648) | 3109.78 (2662.25-3559.77) | 1758711 (1437984-2074273) | 1965.28 (1608.2-2319.11) | -1.78 (-2.1--1.46) |
| Central Europe | 6576425  (5622274-7539949) | 4392.38 (3747.42-5054.05) | 4765687 (3929136-5664253) | 2407.59 (1977.46-2871.77) | -2.09 (-2.16--2.03) |
| Central Latin America | 1419591  (1162254-1690575) | 1601.18 (1312.78-1908.07) | 1913887 (1494155-2357010) | 745.5 (582.97-918.55) | -2.79 (-2.92--2.65) |
| Central Sub-Saharan Africa | 454186  (341045-585176) | 1679.58 (1261.77-2148.36) | 792072 (569588-1036277) | 1127.79 (822.48-1466.09) | -1.27 (-1.42--1.13) |
| Commonwealth High Income | 4966712  (4160191-5782468) | 3417.07 (2855.45-3991.91) | 3126907 (2466955-3796061) | 1255.58 (987.36-1534.84) | -3.31 (-3.4--3.22) |
| Commonwealth Low Income | 2970372  (2400477-3650484) | 3135.78 (2530.24-3852.35) | 4277657 (3197639-5406352) | 1762.21 (1322.75-2225.46) | -1.9 (-1.98--1.82) |
| Commonwealth Middle Income | 20398076  (16331013-24862621) | 3001.08 (2387.78-3665.79) | 28493638 (22448054-34701904) | 1772.96 (1391.58-2159.46) | -1.71 (-1.79--1.64) |
| East Asia | 40205471  (33066159-48005182) | 4712.59 (3877.98-5653.84) | 56296310 (44571479-70508548) | 2598.18 (2057.21-3260.32) | -1.98 (-2.04--1.92) |
| East Asia & Pacific - WB | 54589392  (45729437-64739270) | 4004.53 (3343.5-4741.92) | 76759381 (62050002-92707855) | 2326.53 (1880.56-2813.75) | -1.82 (-1.86--1.78) |
| Eastern Africa | 1572114  (1206925-1977978) | 1987.86 (1531.72-2501.89) | 1916775 (1495910-2393339) | 1021.75 (799.68-1272.03) | -2.43 (-2.53--2.33) |
| Eastern Europe | 10211325  (8930151-11495858) | 3647.5 (3186.21-4118.29) | 8699880 (7270144-10162546) | 2659.92 (2222.21-3105.74) | -1.57 (-2.12--1.03) |
| Eastern Mediterranean Region | 5645115  (4682817-6713226) | 2902.03 (2389.9-3462.43) | 10083982 (8116011-12089657) | 1984.75 (1605.29-2374.58) | -1.35 (-1.47--1.22) |
| Eastern Sub-Saharan Africa | 1610410  (1251620-2055858) | 1892.71 (1466.56-2407.36) | 2032431 (1578611-2575545) | 1012.43 (792.41-1276.98) | -2.25 (-2.34--2.16) |
| Europe | 34592475  (29503431-39843419) | 3452.33 (2941.6-3982.88) | 26664114 (21984172-31444240) | 1900.69 (1564.99-2250.62) | -2.16 (-2.35--1.97) |
| Europe & Central Asia - WB | 35499126  (30274864-40900610) | 3437.32 (2928.88-3964.25) | 27707901 (22857043-32624586) | 1891.51 (1558.61-2234.35) | -2.17 (-2.36--1.97) |
| European Region | 35699403  (30446333-41133816) | 3433.17 (2925.34-3959.69) | 27920267 (23026106-32873933) | 1885.96 (1553.81-2228.87) | -2.17 (-2.37--1.98) |
| High-income Asia Pacific | 4664432  (3818681-5518499) | 2292.57 (1874.01-2719.09) | 4059101 (3275047-4912428) | 1027.8 (807.97-1249.11) | -2.73 (-2.79--2.68) |
| High-income North America | 11307055  (9437180-13217942) | 3394.07 (2836.13-3959.67) | 10351188 (8303129-12508542) | 1705.27 (1352.53-2066.35) | -2.33 (-2.38--2.29) |
| Latin America & Caribbean - WB | 6848818  (5697843-8016715) | 2404.02 (2003.93-2814.62) | 7650673 (6076545-9316632) | 1067.52 (847.64-1300.85) | -2.8 (-2.89--2.71) |
| Limited Health System | 26786574  (21669828-32255346) | 3124.26 (2499.09-3773.12) | 36614438 (29209092-44122935) | 1795.1 (1430.42-2163.72) | -1.83 (-1.88--1.78) |
| Middle East & North Africa - WB | 3444310  (2866517-4034046) | 2554.86 (2121.69-2994.87) | 6848848 (5357249-8262572) | 1781.04 (1395.63-2149.13) | -1.16 (-1.18--1.13) |
| Minimal Health System | 1037968  (784712-1323334) | 1523.38 (1151.91-1942.58) | 1638953 (1217562-2072630) | 1051.08 (779.36-1320.75) | -1.13 (-1.16--1.11) |
| North Africa and Middle East | 5435616  (4535601-6339081) | 2938.5 (2446.2-3437.91) | 9391772 (7463022-11239564) | 1874.78 (1491.07-2241.65) | -1.52 (-1.57--1.47) |
| North America | 11305176  (9435648-13215711) | 3393.28 (2835.46-3958.74) | 10349699 (8301922-12506642) | 1704.9 (1352.26-2065.92) | -2.33 (-2.38--2.29) |
| Northern Africa | 1738605  (1435107-2035094) | 2588.53 (2129.24-3054.19) | 3356531 (2601212-4141591) | 2008 (1554.36-2479.15) | -0.75 (-0.8--0.7) |
| Oceania | 134818  (99207-171300) | 3815.88 (2846.75-4842.04) | 270144 (204295-344140) | 2908.95 (2180.25-3713.16) | -0.95 (-1--0.91) |
| Region of the Americas | 18100830  (15119000-21152042) | 2957.75 (2472.75-3451.76) | 17952194 (14379134-21814479) | 1363.45 (1088.14-1661.83) | -2.64 (-2.7--2.58) |
| South-East Asia Region | 25626221  (20616213-30732550) | 3356.45 (2676.32-4035.31) | 37087542 (29967285-44267430) | 1993.79 (1613.41-2382.53) | -1.68 (-1.72--1.64) |
| South Asia | 21031334  (16893707-25514875) | 3384.42 (2696.85-4115.66) | 29233379 (23125387-35409986) | 1940.77 (1540.64-2356.34) | -1.8 (-1.86--1.74) |
| South Asia - WB | 21422347  (17215390-25987744) | 3347.29 (2669.63-4068.72) | 29715810 (23562265-35969593) | 1922.84 (1527.8-2332.88) | -1.79 (-1.85--1.74) |
| Southeast Asia | 9274525  (7674575-11092594) | 3364.75 (2764.15-4011.42) | 15932992 (13150200-18840787) | 2294.14 (1898.44-2710.66) | -1.3 (-1.37--1.24) |
| Southern Africa | 1341868  (1062516-1646887) | 2684.26 (2132.31-3283.88) | 1932990 (1531773-2364855) | 1743.86 (1379-2127.28) | -1.41 (-1.69--1.14) |
| Southern Latin America | 1265009  (1052809-1471439) | 2696.65 (2241.9-3135.6) | 1139882 (907608-1378210) | 1373.64 (1089.27-1661.92) | -2.06 (-2.13--1.99) |
| Southern Sub-Saharan Africa | 954760  (764781-1157889) | 3088.09 (2481.13-3741.47) | 1234889 (983443-1501581) | 1885.04 (1499.09-2282.26) | -1.59 (-1.94--1.24) |
| Sub-Saharan Africa - WB | 4176279  (3258296-5220908) | 1653.42 (1292.27-2058.65) | 5799087 (4483401-7170985) | 977.24 (757.48-1208.05) | -1.82 (-1.93--1.71) |
| Tropical Latin America | 3384678  (2805160-3933738) | 3408.54 (2824.7-3983.04) | 3540515 (2794869-4322223) | 1349.8 (1065.08-1649.69) | -3.23 (-3.33--3.12) |
| Western Africa | 752194  (559207-953812) | 840.68 (626.85-1063.02) | 1153458 (849553-1464283) | 548.72 (405.62-696.51) | -1.46 (-1.59--1.32) |
| Western Europe | 15811365  (13219980-18509857) | 2915.19 (2429.29-3420.58) | 10788919 (8671983-12985713) | 1339.8 (1066.53-1616.7) | -2.49 (-2.53--2.45) |
| Western Pacific Region | 47706587  (39681462-56502504) | 4103.34 (3419.74-4853.73) | 65467876 (52046615-80683308) | 2320.71 (1846.33-2858.77) | -1.9 (-1.94--1.86) |
| Western Sub-Saharan Africa | 870164  (654126-1114290) | 879.4 (663.17-1121.05) | 1360158 (1004904-1722872) | 577.3 (429.83-730.62) | -1.44 (-1.59--1.29) |
| World Bank High Income | 38809192  (32486703-45353580) | 3121.95 (2610.88-3653.54) | 30989982 (24891862-37391922) | 1483.72 (1183-1791.06) | -2.46 (-2.49--2.43) |
| World Bank Low Income | 3207260  (2506464-3950930) | 1953.53 (1526.82-2395.45) | 4704329 (3586177-5765835) | 1259.9 (965.81-1542.33) | -1.56 (-1.61--1.51) |
| World Bank Lower Middle Income | 35866247  (29637954-42732706) | 3194.01 (2620.17-3805.41) | 52625560 (42905755-62246995) | 2008.09 (1637.46-2379.41) | -1.55 (-1.62--1.48) |
| World Bank Upper Middle Income | 59487631  (50603348-69404963) | 3849.18 (3263.72-4497.69) | 76610906 (61691880-93068419) | 2173.36 (1749.57-2632.84) | -2.02 (-2.13--1.92) |
| **Country** |  |  |  |  |  |
| Afghanistan | 145287  (100120-198986) | 2013.61 (1386.17-2762.85) | 232077 (166194-314138) | 1763.4 (1284.61-2319.58) | -0.5 (-0.85--0.14) |
| Albania | 67842  (55717-79637) | 3257.44 (2674.34-3843.94) | 97813 (77488-119603) | 2334.73 (1847.56-2850.17) | -0.8 (-1.02--0.58) |
| Algeria | 282649  (225693-345679) | 2412.27 (1900.99-2960.83) | 491529 (375472-629610) | 1403.12 (1068.08-1801.7) | -1.89 (-2.02--1.76) |
| American Samoa | 896  (722-1101) | 3339.04 (2682.38-4017.76) | 1500 (1178-1880) | 2853.98 (2241.71-3570.86) | -0.59 (-0.66--0.52) |
| Andorra | 1488  (1094-1959) | 2519.15 (1848.7-3327.32) | 1921 (1312-2574) | 1287.2 (884.78-1726.39) | -2.04 (-2.23--1.85) |
| Angola | 131018  (94768-170770) | 2720.18 (1979.83-3529.49) | 248541 (176583-329558) | 1682.18 (1208.02-2211.69) | -1.59 (-1.77--1.41) |
| Antigua and Barbuda | 553  (429-688) | 1098.35 (850.12-1367.04) | 784 (597-972) | 704.02 (534.57-870.15) | -1.63 (-1.84--1.43) |
| Argentina | 942142  (784087-1097408) | 2896.05 (2411.17-3376.76) | 826100 (654473-1002291) | 1543.59 (1222.04-1872.52) | -1.88 (-1.97--1.78) |
| Armenia | 110689  (96722-124371) | 3780.08 (3294.36-4245.09) | 111646 (94991-129503) | 2621.95 (2229.53-3042.04) | -1.4 (-1.56--1.25) |
| Australia | 452674  (364889-540827) | 2332.05 (1877.43-2784.62) | 345652 (265214-438547) | 874.31 (664.69-1113.64) | -3.17 (-3.26--3.08) |
| Austria | 242652  (201524-287801) | 2269.88 (1883.88-2691.98) | 217242 (172347-262034) | 1368.88 (1083.55-1658.77) | -1.6 (-1.68--1.53) |
| Azerbaijan | 187396  (157994-217717) | 3427.61 (2871.87-3982.1) | 263653 (214413-309735) | 2384.23 (1942.89-2820.64) | -1.22 (-1.47--0.96) |
| Bahamas | 2211  (1733-2718) | 1303.59 (1019.07-1605.35) | 3808 (2686-4905) | 866.08 (610.76-1114.84) | -1.21 (-1.31--1.1) |
| Bahrain | 7425  (5977-9127) | 3749.91 (2984.99-4618.35) | 17586 (13721-22492) | 1678.14 (1310.66-2147.15) | -3.18 (-3.42--2.95) |
| Bangladesh | 2106166  (1715198-2575427) | 4167.53 (3393.89-5106.6) | 2954640 (2227401-3750027) | 2094.01 (1579.56-2661.04) | -2.16 (-2.27--2.05) |
| Barbados | 2528  (1968-3119) | 928.77 (723.95-1146.17) | 2621 (1845-3487) | 535.77 (377.15-711.76) | -2.12 (-2.31--1.92) |
| Belarus | 497142  (422686-567958) | 3879.47 (3298.52-4454.56) | 492789 (387849-604058) | 3226.96 (2540.58-3951.49) | -1.2 (-1.57--0.83) |
| Belgium | 478661  (401386-556058) | 3300.21 (2772.24-3834.29) | 301582 (242826-364598) | 1476.35 (1187.02-1784.75) | -2.56 (-2.64--2.47) |
| Belize | 1122  (899-1335) | 1177.42 (944.7-1397.4) | 2881 (2237-3663) | 889.55 (690.7-1130.23) | -1.1 (-1.43--0.77) |
| Benin | 23742  (17441-31318) | 1106.11 (815.44-1459.44) | 30220 (21153-41393) | 510.09 (360.12-688.59) | -2.68 (-2.83--2.53) |
| Bermuda | 1229  (976-1498) | 1922.14 (1527.51-2335.84) | 1148 (881-1469) | 923.59 (704.16-1183.81) | -2.23 (-2.44--2.02) |
| Bhutan | 4521  (2984-6576) | 1711.21 (1157.92-2477.7) | 6348 (4383-8942) | 1036.66 (719.28-1449.6) | -1.69 (-1.83--1.56) |
| Bolivia (Plurinational State of) | 58373  (44683-74937) | 1667.98 (1268.95-2142.74) | 75405 (56338-104323) | 789.97 (594.6-1089.82) | -2.11 (-2.43--1.8) |
| Bosnia and Herzegovina | 157094  (133186-182063) | 3545.04 (2990.18-4122.29) | 161292 (124512-199224) | 2756.47 (2111.88-3410.04) | -0.84 (-0.98--0.7) |
| Botswana | 25341  (18045-34895) | 4017.97 (2899.06-5479.07) | 32551 (23532-43314) | 1928.53 (1435.73-2561.64) | -2.7 (-3.02--2.38) |
| Brazil | 3335511  (2759093-3872335) | 3438.29 (2843.26-4019.4) | 3445801 (2720818-4205261) | 1344.53 (1061.51-1642.88) | -3.28 (-3.38--3.17) |
| Brunei Darussalam | 4587  (3696-5514) | 3939.8 (3162.25-4745.18) | 6522 (5005-8288) | 1630.73 (1242.49-2059.12) | -2.66 (-2.93--2.38) |
| Bulgaria | 565671  (477030-649013) | 4715.85 (3983.85-5422.28) | 370936 (297614-447504) | 3167.41 (2554.63-3827.23) | -1.61 (-1.76--1.45) |
| Burkina Faso | 40299  (29158-54556) | 815.9 (591.36-1101.45) | 67138 (47149-90886) | 590.25 (420.35-799.53) | -0.99 (-1.17--0.8) |
| Burundi | 73455  (54654-96651) | 2768.45 (2066.29-3640.09) | 59316 (42743-79303) | 965.87 (696.1-1289.33) | -3.66 (-4.14--3.19) |
| Cabo Verde | 1977  (1485-2572) | 938.84 (704.03-1223.03) | 3194 (2287-4264) | 656.17 (474.66-868.01) | -1.54 (-1.85--1.23) |
| Cambodia | 237816  (192031-291269) | 4890.82 (3961.07-5955.47) | 415462 (310065-522001) | 3227.97 (2437.31-4030.53) | -1.6 (-1.76--1.44) |
| Cameroon | 63022  (45860-82655) | 1189.62 (875.99-1558.3) | 120672 (77862-166111) | 765.26 (496.81-1054.88) | -1.49 (-1.85--1.12) |
| Canada | 977247  (822524-1139997) | 3048.97 (2566.02-3553.36) | 816449 (652468-990089) | 1224.99 (968.04-1492.43) | -3.03 (-3.09--2.96) |
| Central African Republic | 42211  (30812-57481) | 2969.65 (2162.39-3990.55) | 58397 (37966-82165) | 1912.68 (1293.98-2632.3) | -1.57 (-1.72--1.41) |
| Chad | 38235  (27048-52218) | 1267.1 (898.7-1732.62) | 68236 (45267-96653) | 1006.43 (671.72-1419.4) | -1 (-1.26--0.74) |
| Chile | 207616  (165793-251873) | 1919.44 (1533.77-2321.64) | 218371 (166453-272635) | 904.28 (685.93-1132.86) | -2.33 (-2.39--2.28) |
| China | 39159330  (32147078-46908741) | 4782.06 (3924.43-5752.16) | 54701549 (43108563-68748854) | 2616.41 (2061.62-3293.19) | -2 (-2.06--1.94) |
| Colombia | 298994  (243362-363149) | 1545.99 (1259.42-1877.79) | 320593 (240138-422947) | 580.01 (433.62-766.04) | -3.61 (-3.76--3.45) |
| Comoros | 4408  (2959-6074) | 2053.31 (1396.28-2794.81) | 5409 (3742-7223) | 1032.72 (720.47-1379.42) | -2.63 (-2.91--2.36) |
| Congo | 24167  (17637-32255) | 2000.59 (1478.23-2656.17) | 45494 (32315-61034) | 1398.45 (1017.9-1824.61) | -1.26 (-1.51--1.01) |
| Cook Islands | 422  (324-525) | 3109.32 (2379.52-3848.41) | 471 (364-611) | 1928.19 (1480.27-2508.45) | -1.61 (-1.71--1.51) |
| Costa Rica | 25920  (20459-31842) | 1423.74 (1124.95-1740.89) | 40358 (30483-51413) | 731.47 (552.8-932.6) | -2.42 (-2.57--2.27) |
| Croatia | 268045  (226302-310886) | 4350.86 (3655.89-5071.26) | 186926 (148957-225257) | 2335.14 (1860.14-2821.11) | -1.84 (-1.91--1.76) |
| Cuba | 289685  (243612-338881) | 2843.2 (2391.5-3326.85) | 351288 (283356-424433) | 1851.43 (1491.5-2238) | -1.53 (-1.64--1.43) |
| Cyprus | 21212  (17324-25185) | 2803.95 (2276.64-3357.88) | 30016 (23652-37366) | 1506.65 (1179.65-1872.74) | -1.95 (-2.01--1.9) |
| Czechia | 613116  (509638-709239) | 4585.43 (3828.17-5313.35) | 382931 (303505-468304) | 1985.23 (1566.66-2444.15) | -2.64 (-2.72--2.56) |
| C?te d'Ivoire | 78927  (57055-104332) | 1532.8 (1107.72-2008.62) | 134136 (90486-183692) | 938.04 (643.3-1273) | -2.17 (-2.56--1.78) |
| Democratic People's Republic of Korea | 624753  (468051-799431) | 3603.59 (2711.47-4549.41) | 1021486 (768333-1276794) | 3004.36 (2262.11-3759) | -0.69 (-0.81--0.56) |
| Democratic Republic of the Congo | 243277  (176188-321538) | 1300.19 (949.58-1708.09) | 419789 (300261-570108) | 883.43 (632.91-1187.66) | -1.19 (-1.31--1.07) |
| Denmark | 353372  (300662-409724) | 4659.57 (3959.08-5401.88) | 204607 (163041-245355) | 1838.67 (1452.89-2230.72) | -3.26 (-3.36--3.16) |
| Djibouti | 4460  (3141-6324) | 2559.46 (1831.29-3567.32) | 13666 (9065-18905) | 1779.46 (1203.99-2456.67) | -1.25 (-1.36--1.14) |
| Dominica | 673  (532-827) | 1168.31 (921.38-1433.02) | 769 (586-1013) | 897.78 (685.53-1183.75) | -0.8 (-1.04--0.55) |
| Dominican Republic | 71010  (54605-88605) | 1831.97 (1409.59-2286.34) | 158152 (119855-210023) | 1551.16 (1177.45-2060.82) | -0.19 (-0.37--0.02) |
| Ecuador | 71992  (57984-88860) | 1268.67 (1017.52-1561.57) | 89878 (65362-115508) | 540.99 (393.92-694.99) | -2.59 (-2.77--2.41) |
| Egypt | 939854  (775018-1109649) | 3116.2 (2550.29-3705.36) | 2000227 (1555165-2475669) | 2858.82 (2231.95-3540.16) | -0.02 (-0.18-0.14) |
| El Salvador | 24345  (18405-30744) | 761.38 (577.1-963.18) | 39741 (28750-52057) | 657.07 (475.79-860.98) | -0.44 (-0.63--0.26) |
| Equatorial Guinea | 5335  (3687-7255) | 2332.57 (1610.7-3178.16) | 6412 (4124-9073) | 990.53 (644.06-1373.44) | -3.09 (-3.66--2.52) |
| Eritrea | 36099  (24248-51595) | 2072.34 (1415.8-2967.53) | 43046 (28105-62064) | 1044.55 (698.09-1484.51) | -2.56 (-2.7--2.43) |
| Estonia | 73391  (62349-84489) | 3665 (3108.04-4226.65) | 34919 (28098-42847) | 1554.65 (1240.17-1918.16) | -3.31 (-3.63--2.99) |
| Eswatini | 6391  (4671-8544) | 2031.07 (1485.57-2695.46) | 9193 (6155-12735) | 1425.42 (968.26-1950.14) | -0.82 (-1.41--0.23) |
| Ethiopia | 336336  (252001-441098) | 1386.5 (1045.08-1810.03) | 205683 (149895-277143) | 405.71 (297.3-546.92) | -4.02 (-4.33--3.7) |
| Fiji | 18807  (14621-23316) | 4178.45 (3205.15-5215.62) | 24764 (18274-33345) | 2788.34 (2060.61-3742.46) | -1.49 (-1.61--1.37) |
| Finland | 160595  (132931-190117) | 2390.55 (1979.65-2832.52) | 104080 (81742-128698) | 1007.15 (785.6-1252.15) | -2.66 (-2.71--2.61) |
| France | 1731778  (1426296-2046767) | 2286.59 (1881.36-2706.27) | 1440754 (1148869-1746526) | 1274.02 (1013.6-1550.6) | -1.75 (-1.78--1.71) |
| Gabon | 8177  (5960-10719) | 1352.61 (994.11-1758.75) | 13439 (9162-18120) | 1089.25 (747.86-1451.13) | -0.71 (-0.79--0.63) |
| Gambia | 8796  (6339-11828) | 2071.3 (1483.78-2756.92) | 13300 (9085-17764) | 1136.66 (784.29-1515.45) | -2.2 (-2.37--2.02) |
| Georgia | 217477  (186405-248010) | 3441.6 (2951.6-3923.27) | 148962 (125412-174151) | 2729.75 (2303.17-3183.98) | -0.7 (-0.9--0.5) |
| Germany | 3520589  (2920826-4171716) | 2978.07 (2467.54-3533.02) | 2471534 (1974410-3010235) | 1516.41 (1207.13-1864.56) | -2.1 (-2.21--1.98) |
| Ghana | 67416  (49343-89084) | 950.94 (700.17-1252.63) | 151239 (107588-204177) | 791.97 (562.53-1054.12) | 0.05 (-0.14-0.24) |
| Greece | 465640  (402354-537671) | 3200.12 (2756.27-3706.76) | 437617 (362548-516081) | 2197.14 (1824.92-2578.05) | -1.2 (-1.26--1.14) |
| Greenland | 2849  (2336-3343) | 7293.72 (6054.44-8521.07) | 2475 (1983-2998) | 3295.88 (2645.35-4003.23) | -2.6 (-2.67--2.54) |
| Grenada | 924  (734-1154) | 1450.87 (1148.96-1807.14) | 1023 (782-1291) | 838.15 (639.71-1057.35) | -1.93 (-2.06--1.8) |
| Guam | 1946  (1578-2373) | 2091.01 (1689.25-2529.27) | 3706 (2968-4586) | 1850.1 (1489.8-2292.18) | -0.08 (-0.24-0.09) |
| Guatemala | 46507  (35132-59166) | 1221.07 (925.59-1570.31) | 69752 (51312-91021) | 604.19 (444.69-786.91) | -2.66 (-2.82--2.5) |
| Guinea | 41626  (30265-55628) | 1189.45 (869.56-1596.29) | 65002 (44391-87546) | 1027.42 (707.02-1383.01) | -0.29 (-0.48--0.1) |
| Guinea-Bissau | 5776  (4028-7873) | 1223.17 (851.9-1667.21) | 9985 (6850-14054) | 1024.8 (717.16-1408.1) | -0.07 (-0.32-0.17) |
| Guyana | 9174  (7252-11498) | 2096.46 (1669.95-2639.33) | 8782 (6150-12046) | 1233.62 (865.85-1688.35) | -1.37 (-1.46--1.29) |
| Haiti | 68251  (51494-87862) | 1804.93 (1367.81-2321.49) | 79010 (53690-109055) | 916.95 (627.45-1268.06) | -2.12 (-2.33--1.91) |
| Honduras | 33815  (26375-42390) | 1507.84 (1169.61-1890.46) | 95945 (73104-124375) | 1455.24 (1120.96-1875.2) | 0.09 (-0.06-0.24) |
| Hungary | 697729  (596710-799615) | 4994.46 (4265.17-5729.7) | 451629 (365745-545174) | 2692.71 (2170.35-3244.06) | -2.17 (-2.27--2.06) |
| Iceland | 7687  (6262-9239) | 2790.22 (2271.86-3345.15) | 6441 (5032-8082) | 1201.95 (935.05-1513.89) | -2.83 (-2.89--2.77) |
| India | 16252735  (12851692-19913679) | 3228.3 (2550.27-3967.3) | 22540404 (17566204-27529015) | 1863.3 (1460.42-2283.96) | -1.74 (-1.82--1.67) |
| Indonesia | 3052190  (2422857-3673729) | 2758.44 (2193.16-3345.66) | 6897786 (5276084-8739279) | 2659.14 (2056.66-3344.52) | -0.01 (-0.16-0.13) |
| Iran (Islamic Republic of) | 530044  (425532-642123) | 1786.89 (1431.1-2168.97) | 963115 (773107-1162921) | 1122.98 (898.18-1356.33) | -1.33 (-1.41--1.25) |
| Iraq | 311624  (249135-380082) | 3716.92 (2962.98-4552.09) | 663880 (493900-855334) | 2580.2 (1914.49-3286.9) | -1.63 (-1.76--1.5) |
| Ireland | 167718  (138439-197300) | 4177.59 (3441.87-4919.09) | 90983 (71861-111242) | 1203.23 (942.25-1478.87) | -4.12 (-4.25--3.99) |
| Israel | 106793  (87398-128025) | 2274.48 (1866.55-2720.16) | 106246 (83415-131757) | 926.52 (727.72-1146.62) | -2.99 (-3.04--2.93) |
| Italy | 2185211  (1823248-2551189) | 2597.97 (2159.26-3037.47) | 1373750 (1092888-1678595) | 1104.8 (877.94-1354.51) | -2.77 (-2.8--2.73) |
| Jamaica | 23434  (18932-28244) | 1372.36 (1111.6-1646.95) | 30159 (21674-40848) | 979.69 (704.32-1324.77) | -1.33 (-1.68--0.99) |
| Japan | 3533271  (2893121-4200773) | 2092.73 (1707.35-2493.98) | 2960654 (2378379-3584619) | 1031.37 (810.23-1248.21) | -2.4 (-2.46--2.34) |
| Jordan | 44898  (36376-54759) | 2967.67 (2399.11-3624.87) | 139698 (109007-174153) | 1611.46 (1252.35-2007.56) | -2.26 (-2.4--2.13) |
| Kazakhstan | 519132  (449693-601444) | 3749.33 (3243.3-4345.28) | 404160 (327794-485913) | 2061.81 (1667.36-2473.15) | -2.7 (-3.22--2.17) |
| Kenya | 137165  (98635-187985) | 1476.92 (1074.05-2024.19) | 264694 (194146-352773) | 975.88 (717.85-1306.47) | -1.46 (-1.71--1.21) |
| Kiribati | 3192  (2534-3978) | 7391.74 (5889.96-9131.59) | 6049 (4560-7755) | 6888.85 (5254.71-8706.16) | -0.35 (-0.54--0.17) |
| Kuwait | 17616  (14359-20944) | 2066.26 (1666.33-2460.91) | 52931 (40365-66364) | 1282.84 (984.07-1620.14) | -1.38 (-1.73--1.02) |
| Kyrgyzstan | 113724  (95355-132575) | 3670.19 (3074.89-4274.37) | 136487 (109554-168055) | 2556.47 (2052.01-3153.03) | -1.2 (-1.54--0.86) |
| Lao People's Democratic Republic | 126227  (93885-158784) | 5596.06 (4199.3-7013.88) | 157689 (116401-198419) | 3178.61 (2353-3967.62) | -1.91 (-1.97--1.86) |
| Latvia | 132048  (113440-151801) | 3795.86 (3256.03-4352.16) | 67645 (55528-81230) | 2081.62 (1704.16-2509.31) | -2.45 (-2.75--2.14) |
| Lebanon | 84512  (66086-104738) | 3691.13 (2872.99-4580.34) | 132069 (103780-162211) | 2201.77 (1731.72-2707.04) | -1.41 (-1.55--1.27) |
| Lesotho | 25577  (18526-34208) | 2865.73 (2076.23-3832.75) | 60000 (44008-77689) | 4823.55 (3521.13-6217.23) | 2.51 (2.06-2.96) |
| Liberia | 12002  (8497-15896) | 964.89 (685.77-1273.75) | 18569 (12411-25763) | 660.5 (441.76-904.01) | -1.48 (-1.62--1.33) |
| Libya | 41588  (32979-51888) | 2004.07 (1595.62-2500.06) | 123689 (91874-158390) | 1996.75 (1495.38-2544.64) | 0.35 (0.19-0.51) |
| Lithuania | 150302  (127366-172408) | 3407.65 (2889.65-3913.02) | 97642 (79973-116977) | 2032.86 (1664.55-2438.58) | -1.78 (-2.05--1.51) |
| Luxembourg | 15591  (12621-18700) | 2975.03 (2408.06-3563.68) | 12683 (9711-15721) | 1261.87 (963.12-1567.56) | -2.84 (-2.9--2.77) |
| Madagascar | 128609  (99072-161826) | 2217.97 (1717.61-2804.24) | 132686 (91725-187954) | 910.89 (636.35-1276.55) | -3.04 (-3.28--2.81) |
| Malawi | 80854  (60696-102769) | 1956.41 (1465.63-2503.78) | 145688 (104831-193430) | 1708.07 (1239.12-2230.25) | -0.75 (-1.06--0.44) |
| Malaysia | 285320  (238771-336372) | 2870.44 (2397.77-3379.88) | 560695 (464357-658985) | 1893.7 (1562.51-2241.87) | -1.45 (-1.58--1.32) |
| Maldives | 3969  (3263-4741) | 4271.37 (3543.65-5130.03) | 5074 (3910-6299) | 1325.59 (1036.21-1648.76) | -4.22 (-4.47--3.97) |
| Mali | 38864  (27473-51950) | 899.71 (635.15-1192.57) | 89274 (63187-119751) | 937.45 (665.75-1259.17) | 0.55 (0.37-0.73) |
| Malta | 11450  (9349-13648) | 2666.61 (2174.94-3185.15) | 9218 (7180-11431) | 1170.8 (911.97-1447.82) | -2.68 (-2.77--2.58) |
| Marshall Islands | 845  (661-1052) | 4242.13 (3334.23-5312.63) | 1714 (1242-2274) | 3794.47 (2758.85-4998.3) | -0.27 (-0.35--0.19) |
| Mauritania | 16207  (11757-21383) | 1429.75 (1038.73-1878.98) | 16880 (11260-22813) | 678.34 (451.9-908.31) | -2.55 (-2.78--2.32) |
| Mauritius | 24760  (20727-29203) | 3124.34 (2601.79-3695.36) | 29482 (24421-34682) | 1617.27 (1340.63-1895.28) | -2.26 (-2.57--1.94) |
| Mexico | 755518  (614483-902240) | 1723.09 (1400.11-2064.5) | 946735 (738036-1184910) | 727.88 (566.98-908.11) | -3.11 (-3.28--2.94) |
| Micronesia (Federated States of) | 3196  (2455-4136) | 5832.59 (4468.05-7546.19) | 4327 (3232-5595) | 4828.6 (3616.64-6185.2) | -0.61 (-0.65--0.57) |
| Monaco | 1644  (1233-2049) | 2797.9 (2108.49-3475.83) | 1694 (1335-2144) | 2093.53 (1638.37-2684.36) | -0.89 (-1.01--0.78) |
| Mongolia | 42203  (33588-50964) | 3754.38 (2995.15-4532.07) | 82068 (64104-100826) | 2966.35 (2302.93-3660.36) | -0.9 (-1.15--0.65) |
| Montenegro | 25286  (21546-29058) | 3895.81 (3310.28-4490.39) | 32676 (26675-39297) | 3410.83 (2784.14-4092.87) | -0.44 (-0.7--0.17) |
| Morocco | 325318  (262918-391617) | 2089.29 (1693.12-2517.26) | 469626 (352410-590847) | 1266.94 (951.18-1597.27) | -1.71 (-1.88--1.54) |
| Mozambique | 113135  (81711-151617) | 1666.58 (1214.39-2213.37) | 210143 (153935-283634) | 1534.43 (1135.24-2061.85) | 0.4 (0.16-0.64) |
| Myanmar | 1687678  (1274395-2118829) | 6992.99 (5329.98-8763) | 1241787 (930850-1590376) | 2535.64 (1902.46-3234.29) | -3.61 (-3.75--3.48) |
| Namibia | 19855  (14825-25687) | 3016.25 (2262.37-3882.52) | 25330 (17731-33574) | 1778.9 (1271.17-2307.02) | -2.03 (-2.39--1.66) |
| Nauru | 436  (329-544) | 7404.25 (5637.08-9263.01) | 466 (341-599) | 6264.03 (4589.68-8101.03) | -0.68 (-0.99--0.37) |
| Nepal | 558847  (422599-736228) | 5715.02 (4325-7493.11) | 623680 (458905-794863) | 2739.55 (2038.07-3493.74) | -2.5 (-2.66--2.33) |
| Netherlands | 645482  (547425-743651) | 3350.28 (2844.88-3872.3) | 477435 (386410-572709) | 1447.46 (1160.9-1743.66) | -2.72 (-2.79--2.65) |
| New Zealand | 110099  (89076-131459) | 2865.95 (2319.94-3425.47) | 90159 (71350-111617) | 1153.43 (904.87-1443.53) | -3.08 (-3.26--2.9) |
| Nicaragua | 16273  (12409-20707) | 935.82 (721.5-1188.31) | 33582 (24965-43736) | 635.99 (471.99-822.91) | -0.98 (-1.16--0.81) |
| Niger | 22096  (15158-30716) | 655.22 (463.83-902.58) | 39743 (26398-55553) | 428.67 (287.09-585.65) | -1.4 (-1.48--1.31) |
| Nigeria | 289078  (203697-393260) | 596.57 (421.88-803.04) | 353164 (241248-490754) | 321.38 (223.74-443.34) | -2.23 (-2.48--1.97) |
| Niue | 64  (48-82) | 3080.37 (2330.33-3977.21) | 58 (44-78) | 2664.29 (2008.21-3599.06) | -0.72 (-0.81--0.63) |
| North Macedonia | 84884  (69801-99745) | 4307.22 (3533.92-5053.73) | 99378 (78068-121127) | 3036.91 (2388.77-3716.4) | -1.33 (-1.58--1.08) |
| Northern Mariana Islands | 834  (621-1081) | 3312.16 (2570.81-4195.39) | 1438 (1162-1754) | 2413.55 (1940.2-2933.46) | -1 (-1.1--0.9) |
| Norway | 164715  (134545-196493) | 2694.72 (2208.99-3197.71) | 89092 (70005-111342) | 979.61 (760.58-1231.84) | -3.56 (-3.7--3.43) |
| Oman | 15163  (11106-20085) | 1789.42 (1289.01-2408.07) | 23495 (17189-30277) | 823.21 (603.48-1068.73) | -2.19 (-2.3--2.08) |
| Pakistan | 2109065  (1659406-2568634) | 3612.81 (2818.93-4399.13) | 3108307 (2295780-4089675) | 2354.57 (1748.23-3082.82) | -1.69 (-1.98--1.39) |
| Palau | 383  (287-492) | 3529.21 (2666.94-4561.68) | 714 (527-940) | 2839.13 (2098.73-3725.5) | -0.73 (-0.79--0.68) |
| Palestine | 26257  (20140-33069) | 2979.31 (2285.23-3738.77) | 54122 (42619-65881) | 1939.03 (1523.92-2378.45) | -1.59 (-1.79--1.4) |
| Panama | 16934  (13507-20906) | 1106.43 (881.36-1372.11) | 23474 (17191-31092) | 530.82 (388.47-702.56) | -2.65 (-2.8--2.5) |
| Papua New Guinea | 77588  (53635-103821) | 3565.18 (2481.93-4811.44) | 178676 (128063-238466) | 2762.19 (1977.42-3707.53) | -0.9 (-0.95--0.85) |
| Paraguay | 49168  (39657-60823) | 2143.64 (1735.43-2639.14) | 94714 (67680-125244) | 1590.39 (1138.53-2102.99) | -0.99 (-1.08--0.91) |
| Peru | 99635  (75818-125024) | 768.02 (582.4-965) | 169010 (124598-232885) | 487.5 (358.95-670.87) | -1.85 (-2.19--1.51) |
| Philippines | 1249441  (1027036-1503587) | 3759.16 (3077.66-4523.66) | 2348780 (1809319-2911631) | 2591.76 (1988.38-3207.79) | -1.17 (-1.32--1.02) |
| Poland | 2203407  (1894586-2527462) | 5054.57 (4348.92-5797.44) | 1479459 (1219427-1775702) | 2249.9 (1847.62-2708.23) | -2.65 (-2.73--2.58) |
| Portugal | 292729  (237859-346669) | 2205.79 (1788.03-2620.68) | 208975 (163778-253687) | 1103.93 (859.66-1344.43) | -2.31 (-2.38--2.24) |
| Puerto Rico | 50686  (39191-64302) | 1412.43 (1091.21-1794.13) | 45838 (34025-61296) | 767.26 (567.93-1031.07) | -2.28 (-2.41--2.15) |
| Qatar | 4180  (3164-5398) | 2493.1 (1907.22-3204.67) | 18043 (12756-24008) | 997.96 (705.14-1322.94) | -3.18 (-3.6--2.76) |
| Republic of Korea | 1072376  (866239-1270542) | 3347.76 (2680.73-3994.4) | 1038683 (820837-1264261) | 1148.73 (900.4-1407.89) | -3.72 (-3.83--3.62) |
| Republic of Moldova | 149928  (124814-177480) | 3249.91 (2707.04-3856.79) | 143085 (118414-169372) | 2501.17 (2073.61-2953.31) | -0.96 (-1.26--0.65) |
| Romania | 1012824  (845631-1170255) | 3635.06 (3028.16-4216.99) | 778068 (614417-932667) | 2470.89 (1951.21-2963.09) | -1.81 (-2.03--1.6) |
| Russian Federation | 6626659  (5807756-7463117) | 3624 (3173.73-4085.77) | 6053299 (5047380-7102033) | 2686.95 (2246.44-3146.82) | -1.53 (-2.14--0.91) |
| Rwanda | 126714  (93839-164853) | 4156.21 (3086.25-5378.79) | 126470 (90088-168703) | 1945.3 (1406.7-2582.87) | -3.46 (-3.85--3.08) |
| Saint Kitts and Nevis | 437  (342-546) | 1275.73 (998.56-1586.49) | 478 (346-632) | 641.28 (463.97-841.89) | -2.49 (-2.66--2.32) |
| Saint Lucia | 1458  (1159-1782) | 1667.26 (1323.28-2037.67) | 1984 (1468-2587) | 816.61 (604.55-1065.82) | -2.63 (-2.78--2.47) |
| Saint Vincent and the Grenadines | 804  (633-980) | 1136.77 (896.58-1381.73) | 1236 (954-1563) | 861.17 (662.08-1088.85) | -1.06 (-1.17--0.95) |
| Samoa | 3586  (2801-4442) | 3917.6 (3051.42-4848.68) | 5163 (3934-6475) | 3291.24 (2525.23-4101.57) | -0.69 (-0.77--0.6) |
| San Marino | 654  (524-809) | 1976.62 (1569.65-2445.93) | 547 (376-762) | 874.13 (599.17-1215.38) | -2.14 (-2.33--1.96) |
| Sao Tome and Principe | 478  (344-632) | 709.05 (511.1-934.42) | 897 (651-1219) | 690.83 (506.33-918.87) | -0.29 (-0.62-0.04) |
| Saudi Arabia | 114809  (79936-155686) | 1534.01 (1072.06-2056.86) | 473067 (346169-619661) | 1433.65 (1057.74-1842.61) | 0.01 (-0.18-0.21) |
| Senegal | 55165  (41164-71385) | 1463.03 (1095.23-1895.38) | 69357 (48312-90807) | 763.48 (530.5-1011.91) | -2.2 (-2.32--2.08) |
| Serbia | 469157  (393473-541465) | 4038.02 (3397.31-4701.27) | 425766 (343019-515302) | 2867.97 (2306.68-3471.62) | -1.34 (-1.61--1.08) |
| Seychelles | 1799  (1471-2103) | 3216.48 (2627.34-3756.12) | 2291 (1841-2792) | 1880 (1510.61-2298.16) | -1.68 (-1.83--1.53) |
| Sierra Leone | 37140  (27215-48715) | 1657.86 (1207.54-2170.35) | 46237 (32564-62356) | 1006.51 (711.08-1349.61) | -1.37 (-1.55--1.19) |
| Singapore | 54198  (43259-64869) | 2261.83 (1812.6-2699.93) | 53243 (41332-65957) | 607.41 (473.47-753.81) | -4.14 (-4.19--4.08) |
| Slovakia | 239378  (202161-276651) | 4075.59 (3449.47-4700.79) | 177477 (141368-216361) | 1978.05 (1570.53-2416.88) | -2.23 (-2.31--2.14) |
| Slovenia | 66823  (54370-79199) | 2732.69 (2222.44-3240.86) | 51970 (40239-64524) | 1379 (1051.79-1721.23) | -2.2 (-2.28--2.11) |
| Solomon Islands | 8719  (5699-11536) | 5459.53 (3683.67-7124.25) | 20396 (15174-26320) | 4797.06 (3591.52-6194.24) | -0.26 (-0.46--0.05) |
| Somalia | 81506  (53872-120592) | 2517.57 (1709.56-3608.18) | 138528 (91591-199936) | 1639.52 (1109.28-2308.56) | -1.52 (-1.59--1.45) |
| South Africa | 766512  (619407-923241) | 3203 (2586.63-3862.85) | 867635 (691295-1051908) | 1670.84 (1334.94-2025.48) | -2.18 (-2.5--1.85) |
| South Sudan | 55219  (38061-80095) | 1991.12 (1370.43-2897.55) | 61821 (40921-85201) | 1333.33 (895.93-1832.65) | -1.56 (-1.81--1.3) |
| Spain | 1380114  (1165378-1587035) | 2690.19 (2265.25-3102.77) | 1067551 (870199-1270383) | 1266.53 (1035.35-1518.99) | -2.42 (-2.51--2.32) |
| Sri Lanka | 241756  (197457-293002) | 2192.58 (1768.53-2684.39) | 245280 (158702-339550) | 918.46 (599.5-1264.42) | -2.74 (-2.87--2.6) |
| Sudan | 265840  (200242-338964) | 2614.93 (1966.76-3333.84) | 363214 (256628-507392) | 1623.05 (1181.17-2258.17) | -1.71 (-1.79--1.63) |
| Suriname | 6361  (5185-7573) | 2304.76 (1866-2759.63) | 9313 (6869-12478) | 1397.53 (1028.78-1875.47) | -1.77 (-1.99--1.55) |
| Sweden | 294105  (236011-355255) | 2112.52 (1687.46-2550.89) | 201185 (153884-253987) | 1014.22 (772.77-1294.92) | -2.28 (-2.34--2.22) |
| Switzerland | 239814  (194916-286704) | 2472.61 (2011.04-2955.18) | 181443 (142839-222937) | 1109.52 (865.36-1365.42) | -2.51 (-2.59--2.43) |
| Syrian Arab Republic | 207141  (160065-258147) | 3536.93 (2738.64-4404.73) | 330768 (236747-437289) | 2337.98 (1678.81-3071.92) | -1.67 (-1.82--1.52) |
| Taiwan (Province of China) | 421388  (349063-492128) | 2540.09 (2104.88-2963.21) | 573276 (458168-679279) | 1416.3 (1134.06-1681.71) | -1.92 (-1.99--1.85) |
| Tajikistan | 92520  (75524-111136) | 3176.66 (2595.79-3801.19) | 84672 (62047-113147) | 1277.52 (940.48-1706.57) | -3.17 (-3.42--2.91) |
| Thailand | 1083097  (871544-1295204) | 2907.96 (2332.85-3466.61) | 1528828 (1122611-2051615) | 1448.05 (1068.25-1935.08) | -2.71 (-2.87--2.56) |
| Timor-Leste | 10508  (7546-14226) | 3122.34 (2372.97-4184.01) | 22228 (16442-29081) | 2562.86 (1890.78-3335.6) | -0.54 (-0.75--0.33) |
| Togo | 29290  (21200-38786) | 2079.23 (1506.42-2741.94) | 62897 (43088-87124) | 1368.31 (938.54-1890.99) | -1.4 (-1.53--1.27) |
| Tokelau | 43  (32-56) | 3232.44 (2351.79-4233.17) | 35 (27-47) | 2457.9 (1848.93-3274.11) | -1.03 (-1.08--0.97) |
| Tonga | 2049  (1605-2571) | 3604.72 (2809.47-4528.57) | 2401 (1804-3119) | 2938.31 (2211.19-3796.38) | -0.71 (-0.84--0.59) |
| Trinidad and Tobago | 19040  (15438-22760) | 2183.33 (1771.91-2613.61) | 22843 (15931-30426) | 1190.62 (831.79-1585.19) | -2.38 (-2.6--2.16) |
| Tunisia | 132990  (108905-158504) | 2608.28 (2136.76-3130.59) | 254580 (193110-341092) | 1880.97 (1424.18-2511.97) | -1.32 (-1.42--1.22) |
| Turkmenistan | 74874  (63427-87052) | 3543.55 (3008.37-4118.71) | 95430 (71282-123451) | 2075.95 (1545.09-2677.6) | -2.32 (-2.67--1.97) |
| Tuvalu | 389  (300-499) | 5183.76 (3996.67-6626.27) | 431 (331-549) | 3870.33 (2969.08-4924.05) | -0.89 (-0.94--0.83) |
| Türkiye | 1699909  (1383275-2009477) | 4493.7 (3647.05-5302.77) | 2068002 (1611601-2600442) | 2142.33 (1666.66-2692.08) | -2.62 (-2.86--2.38) |
| Uganda | 91547  (65140-125521) | 1299.79 (938.79-1762.62) | 143568 (99835-196538) | 828.85 (580.51-1121.75) | -2.42 (-2.89--1.96) |
| Ukraine | 258185  (2232467-2937305) | 3673.21 (3173.74-4187.34) | 1810502 (1240375-2486689) | 2567.24 (1754.16-3524.9) | -1.75 (-2.22--1.29) |
| United Arab Emirates | 16488  (11971-21866) | 2335.86 (1725.39-3120.51) | 73756 (52535-98594) | 1142.97 (822.94-1511.88) | -1.69 (-2.11--1.27) |
| United Kingdom | 3308675  (2777997-3836887) | 3895.3  (3251.31-4533.26) | 1742824  (1373156-2115808) | 1460.6  (1147.76-1781.23) | -3.26 (-3.36--3.16) |
| United Republic of Tanzania | 277651  (206750-365105) | 2304.18 (1726.96-3006.02) | 386217 (282381-519241) | 1291.31 (941.17-1747.11) | -2.32 (-2.48--2.16) |
| United States of America | 10326700 (8604004-12064765) | 3433.76 (2864.63-4002.64) | 9532102 (7630777-11512956) | 1762.46 (1397.46-2134.38) | -2.27 (-2.31--2.22) |
| United States Virgin Islands | 1248  (917-1684) | 1323.57 (975.9-1782.36) | 1191 (859-1615) | 735.94 (529.55-994.23) | -1.85 (-1.99--1.71) |
| Uruguay | 115190  (97394-131491) | 3119.43 (2635.53-3574.29) | 95348 (76884-112224) | 1978.33 (1593.26-2331.08) | -1.66 (-1.74--1.59) |
| Uzbekistan | 202733  (162673-243364) | 1647.25 (1315.54-1972.47) | 431633 (339305-528828) | 1426.8 (1119.64-1750.96) | -0.59 (-1.04--0.14) |
| Vanuatu | 2822  (2140-3652) | 3789.29 (2883.34-4841.43) | 5705 (4412-7186) | 2724.69 (2101.96-3401.58) | -1.3 (-1.4--1.19) |
| Venezuela (Bolivarian Republic of) | 201285  (160522-240379) | 1888.98 (1521.83-2257.94) | 343707 (242892-458208) | 1106.77 (785.26-1476.81) | -2.1 (-2.26--1.94) |
| Viet Nam | 1256547  (954821-1607346) | 3053.51 (2321.8-3911.9) | 2455386 (1877890-3005916) | 2332.34 (1789.24-2820.27) | -0.88 (-0.91--0.85) |
| Yemen | 219052  (161072-289571) | 3845.09 (2849.1-5072.83) | 437539 (307348-587004) | 2664.38 (1874.01-3507.35) | -1.39 (-1.48--1.3) |
| Zambia | 62101  (47412-79950) | 2002.51 (1523.57-2566.85) | 93727 (64544-124160) | 1197.51 (828.92-1561.52) | -2.4 (-2.75--2.04) |
| Zimbabwe | 111083  (84128-144489) | 2568.54 (1971.55-3363.83) | 240181 (171456-315603) | 2951.49 (2107.58-3830.87) | 0.8 (0.26-1.35) |
